# Exploring the microbiota-gut-brain axis: impact on brain structure and function

**DOI:** 10.3389/fnana.2025.1504065

**Published:** 2025-02-12

**Authors:** Lidya K. Yassin, Mohammed M. Nakhal, Alreem Alderei, Afra Almehairbi, Ayishal B. Mydeen, Amal Akour, Mohammad I. K. Hamad

**Affiliations:** ^1^Department of Anatomy, College of Medicine and Health Sciences, United Arab Emirates University, Al Ain, United Arab Emirates; ^2^Department of Pharmacology and Therapeutics, College of Medicine and Health Sciences, United Arab Emirates University, Al Ain, United Arab Emirates

**Keywords:** microbiota-gut-brain axis, short-chain fatty acid, blood–brain barrier, brain morphology, microbial interventions, neuroplasticity

## Abstract

The microbiota-gut-brain axis (MGBA) plays a significant role in the maintenance of brain structure and function. The MGBA serves as a conduit between the CNS and the ENS, facilitating communication between the emotional and cognitive centers of the brain via diverse pathways. In the initial stages of this review, we will examine the way how MGBA affects neurogenesis, neuronal dendritic morphology, axonal myelination, microglia structure, brain blood barrier (BBB) structure and permeability, and synaptic structure. Furthermore, we will review the potential mechanistic pathways of neuroplasticity through MGBA influence. The short-chain fatty acids (SCFAs) play a pivotal role in the MGBA, where they can modify the BBB. We will therefore discuss how SCFAs can influence microglia, neuronal, and astrocyte function, as well as their role in brain disorders such as Alzheimer’s disease (AD), and Parkinson’s disease (PD). Subsequently, we will examine the technical strategies employed to study MGBA interactions, including using germ-free (GF) animals, probiotics, fecal microbiota transplantation (FMT), and antibiotics-induced dysbiosis. Finally, we will examine how particular bacterial strains can affect brain structure and function. By gaining a deeper understanding of the MGBA, it may be possible to facilitate research into microbial-based pharmacological interventions and therapeutic strategies for neurological diseases.

## 1 Introduction

The gut microbiota refers to the collective of microorganisms that inhabit the gastrointestinal tract (GI). The gut microbiota constitutes approximately 1–2 kg of the adult human body (Forsythe and Kunze, 2013; Toda et al., 2019), which is equivalent in weight to that of a normal adult brain. A multitude of physiological processes within the human body can be ascribed to the gut microbiota, with the maturation and development of the central nervous system (CNS) representing a particularly pivotal role (Liang et al., 2018; Cryan et al., 2019; Socała et al., 2021; Loh et al., 2024), as well as the development and modulation of the immune response (Zhang et al., 2021; Castillo-Álvarez and Marzo-Sola, 2022). In contrast to the brain, the gut microbiota is susceptible to direct intervention through the administration of prebiotics, probiotics, synbiotics, and antibiotics, and is amenable to modification by lifestyle factors. A substantial body of research has indicated that the microbiota may be involved in regulating brain morphology. For example, germ-free (GF) animals have demonstrated brain abnormalities in the absence of microbiota, as evidenced by studies (Sudo et al., 2004; Gareau et al., 2011; Heijtz et al., 2011; Neufeld et al., 2011; Clarke et al., 2013). Moreover, alterations in behaviour have been observed in animals administered specific strains of bacteria (Bercik et al., 2011; Bravo et al., 2011; Savignac et al., 2014; Desbonnet et al., 2015; Jarosz et al., 2024; Wlaź et al., 2024). Moreover, evidence indicates that exposure to a single microbial strain can confer protection against certain neurological disorders and systemic immune alterations. The findings of this study lend support to the hypothesis that microbe-based interventions may prove beneficial in the treatment of neurological disorders.

A variety of pathways facilitate the transmission of signals generated in the gut to the brain. The microbiota in the gut can communicate with the brain in several ways. Nevertheless, further research is necessary to gain a comprehensive understanding of the influence that bacteria in the GI tract exert on the brain and behaviour (Guzzetta et al., 2022; Kasarello et al., 2023). The release of cytokines by immune cells into the circulation represents the primary mode of immune communication. Moreover, pathogen-associated or damage-associated molecular patterns may enter the circulation and affect the functioning of internal organs and the gut microbiota. Since both pathogen-associated molecular patterns (PAMPs), derived from microorganisms, and damage-associated molecular patterns (DAMPs), released from stressed host cells, are identified by the immune system through pattern recognition receptors (PRRs) present by dendritic cells and macrophages. Such recognition contributes to immune dysregulation and intestinal permeability, which might collectively impact the gut microbiota by reducing microbial diversity, encouraging detrimental pathobionts, and hindering the microbiota’s capability to effectively regulate immune responses (Stringer, 2024). Endocrine communication represents the most expansive form of communication, encompassing the hypothalamic–pituitary–adrenal axis (HPA). The primary mode of neural communication is through direct anatomical connections established by the vagus nerve or indirect connections facilitated by the enteric nervous system (ENS). The development of novel techniques for studying the microbiome has enabled a more profound comprehension of the interrelationship between neurological disorders and the gut microbiota (Zhu et al., 2021; Loh et al., 2024). A variety of techniques have been developed for the study of MGBA. These include the GF mouse model, antibiotic-induced dysbiosis, fecal microbiota transplantation (FMT), probiotics, prebiotics, and synbiotics. There is substantial evidence indicating that microbes within the gut microbiome play a role in alterations to brain morphology. MGBA has been demonstrated to exert influence over several processes within the CNS, including neurogenesis, the growth of neuronal and microglia dendrites, axon growth, myelination, the structure and permeability of the blood–brain barrier (BBB), and the structure and function of synapses. These topics will be discussed in detail in this review.

The microbiota exerts an influence on the brain by producing short-chain fatty acids (SCFAs). The primary metabolites generated in the colon through bacterial fermentation of dietary fibres and resistant starch are acetate, propionate, and butyrate. Additionally, minor metabolites such as lactate, valerate, and formate are produced (Pascale et al., 2018). There is a considerable body of evidence that the MGBA can influence neuroplasticity in the brain (Leung and Thuret, 2015; Murciano-Brea et al., 2021; Tang et al., 2021; Salami and Soheili, 2022; Sarubbo et al., 2022; Damiani et al., 2023; Lu et al., 2024). The gut microbiota plays a significant role in the conversion of ingested food into nutrients. Consequently, the microbiota may be regarded as a “filter” and a “sensor” for exogenous compounds that enter the body (Clarke et al., 2014). The impact of microbiota alterations on brain plasticity can be exerted through a multitude of mechanisms, including the regulation of gene expression, the production of neuroactive molecules, and the modulation of microglial activity. This review will examine the modulation of brain plasticity by gut microbiota through neurotrophic factors and the impact of aging-related alterations in gut microbiota on the elderly. The objective of this review is to provide a comprehensive overview of how the field of MGBA has increased understanding of the influence of MGBA on brain structure and function. This is crucial for advancing research into microbial-based interventions and therapeutic strategies for neurological diseases.

## 2 Structures and functions of the brain

The blood–brain barrier (BBB) is a structural and biochemical barrier that protects the brain by regulating which metabolites and nutrients can enter the brain from the blood or exit into circulation (Schiera et al., 2024). Its genesis is controlled by brain cells that interact with brain capillary endothelial cells, including pericytes, glial cells, neurons, and astrocytes (Schiera et al., 2024). Both astrocytes and microglia support synaptic development and remodelling in a healthy brain (Vainchtein and Molofsky, 2020). However, they differ in their physiological functions. Astrocytes surround most neuronal synapses, contributing to the formation of the brain’s borders and vasculature. Meanwhile, the role of microglia during development appears to be phagocytic. They engulf apoptotic neuronal corpses and phagocytose synapses (Sofroniew, 2014). Following an injury, astrocytes enlarge to rebuild the BBB, and maintain its structural integrity, while microglia boost their phagocytic activity to clear debris (Peri and Nüsslein-Volhard, 2008). Also, microglia are essential for normal brain myelination. Despite being the most abundant glial cell type in CNS, less is known about astrocytes’ role in this process (Molina-Gonzalez and Miron, 2019). Another open question is whether transplanting astrocytes can restore healthy myelination in progressive diseases. The examples above represent a small subset of astrocyte and microglia roles, including myelination formation, angiogenesis, and BBB regulation. Future research focusing on structural and functional changes in astrocytes and microglia caused by disruptions in the gut microbiome may reveal novel therapeutic interventions for brain disorders.

### 2.1 MGBA modulation of structural and functional domains of the central nervous system

**Figure 1:**
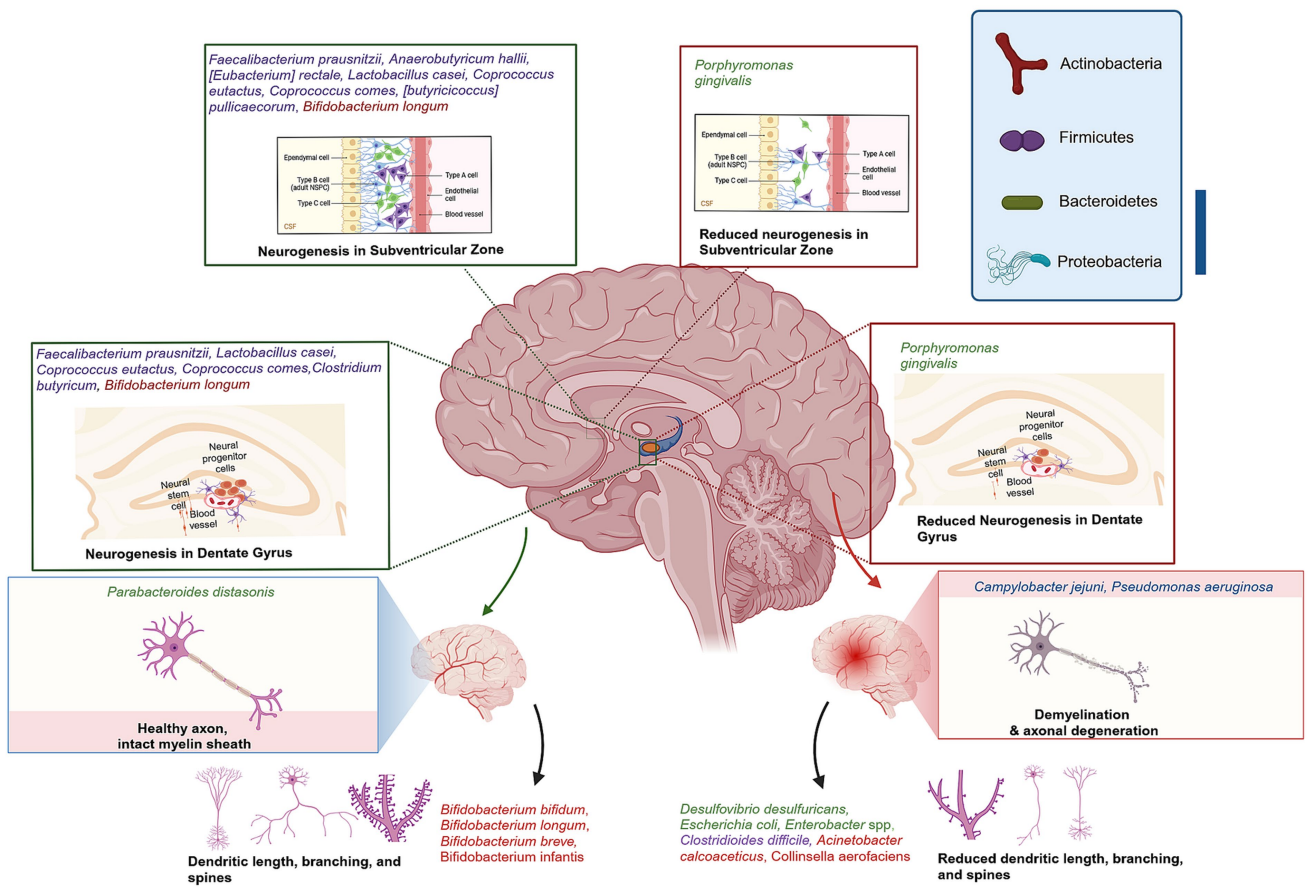
Gut-Brain axis association with brain morphology alterations. Green highlighted bacteria have shown the ability to ameliorate DG neurogenesis, CA3-CA1 synaptic activity, hippocampal BDNF–TrkB signalling, improve hippocampus neurogenesis, myelination gene regulation, maintain dendritic spine density and branching. Red highlighted bacteria were reported to reduce synaptic strength and plasticity, induce neuroinflammation, dendritic spine loss, and decreased dendritic arborization or branching. Created with BioRender.com.

Accumulating evidence suggests that microbes within the gut microbiome are involved in brain morphology alterations. First, research in GF animals demonstrated that the brain morphology is impacted when the microbiota is absent (Hegstrand and Hine, 1986; Sudo et al., 2004; Gareau et al., 2011; Heijtz et al., 2011; Neufeld et al., 2011; Clarke et al., 2013). Secondly, animals that received particular strains of bacteria observed changes in different brain regions (Mckernan et al., 2010; Bercik et al., 2011; Savignac et al., 2014; Desbonnet et al., 2015). Also, human genomic studies on these strains and the brain validated the possible applicability of the findings (Tillisch et al., 2013; Allen et al., 2016; Pinto-Sanchez et al., 2017). Furthermore, population-based research on individuals affected by infection, particularly in Canada, has shown changes in brain structure and overall microbiota composition (Thabane et al., 2010). Finally, preclinical studies have demonstrated long-term impacts on the brain, spinal cord, and ENS from antibiotic exposure or chronic bacterial infection during early life or adulthood as a result of gut dysbiosis (Verdu et al., 2008; O’Mahony et al., 2014). The gut microbiota can influence neurogenesis, myelination, dendritic morphology, microglia morphology, BBB structure and permeability, synapse structure and function (Figure 1).

#### 2.1.1 Neurogenesis

Hippocampal neurogenesis is decreased significantly in the GF mice model or dysbiosis induced by vancomycin in C57BL/6 mice (Möhle et al., 2016; Sawada et al., 2018). However, *Lactobacillus casei* has been demonstrated to ameliorate dentate gyrus (DG) neurogenesis, CA3-CA1 synaptic activity, and hippocampal brain-derived neurotrophic factor-Tropomyosin receptor kinase B (BDNF–TrkB) signaling when impaired by dysbiosis of two-week antibiotic cocktail administration (Guida et al., 2018) and increase serotonin mRNA levels in juvenile rats DG (Barrera-Bugueño et al., 2017). While, colonization of GF mice with control mice microbiota has demonstrated increased levels of neurogenesis, interestingly, the same colonization of the GF mice lacking TPH1, has eliminated serotonin synthesis and decreased Nestin+ neural precursors, hence reducing levels of neurogenesis (De Vadder et al., 2018). In addition, adult neurogenesis is significantly influenced by the microbiome (Ogbonnaya et al., 2015; Sawada et al., 2018). Butyrate, a metabolite produced by gut bacteria, has been shown to elevate hippocampal neurogenesis in pigs. It is therefore hypothesized that butyrate producing bacteria such as *Faecalibacterium prausnitzii, Anaerobutyricum hallii, [Eubacterium] rectale, Lactobacillus casei, Coprococcus eutactus, Coprococcus comes, [butyricicoccus] pullicaecorum*, and *Clostridium butyricum* can influence neurogenesis via gut-brain axis (Hébuterne, 2003; Miquel et al., 2013; Martín et al., 2018; Fusco et al., 2023).

#### 2.1.2 Myelination

Myelination is the formation of the myelin sheath surrounding the nerve, which enhances its conductivity (Hughes and Appel, 2016). Emerging evidence supports the link between intestinal microbiota and myelin formation, as GF C57BL/6 mice have displayed decreased expression of myelin basic protein (Lu et al., 2018), while another animal study has reported hypermyelination, resulting in thicker myelin sheaths in the prefrontal cortex (PFC) and axon functional impairment (Hoban et al., 2016). These findings suggest the importance of intestinal bacteria in myelination gene regulation. The findings are supported by evidence from a mouse model of Huntington’s disease. When compared with a control group an abnormal increase in axon myelin thickness and impaired white matter integrity were recorded in the corpus callosum, as well as downregulation of myelin-related proteins and mature oligodendrocytes (Radulescu et al., 2019). Furthermore, GF B6-deficient Wistar rats exhibited a significantly higher incidence of irregular myelin splitting when compared with conventional B6-deficient rats in the peripheral nervous system (Sumi et al., 1977). The abundance of the *Akkermansia* genus has been found to be correlated with autoimmune encephalomyelitis and myelin damage in a mouse model of multiple sclerosis (MS) (Lee et al., 2011). These findings are consistent with those of a study that observed a similar pattern in MS patients. Specifically, the study found that MS patients exhibited a high abundance of *Akkermansia muciniphila*, *Acinetobacter calcoaceticus*, and low levels of *Parabacteroides distasonis,* a strain linked with anti-inflammatory activity. Indeed, these results were compared with those of healthy human samples (Cekanaviciute et al., 2017).

#### 2.1.3 Dendritic morphology

During early development, dendritic growth is regulated by both cell-intrinsic programs and extrinsic factors that regulate various aspects of dendritic development. It was always believed that dendritic growth is intrinsically determined. However, over the past 2 decades, many studies have shown that the dendritic growth process is remarkably responsive to extrinsic factors, influencing local and global mechanisms of dendrite development (Valnegri et al., 2015; Hamad et al., 2023). The extrinsic factors include neurotransmitters and neurotrophins. Morphological modifications of dendrites could significantly influence their signal integration, neuronal stimulation, and their overall function (Cline, 2001; Inglis et al., 2002; Jan and Jan, 2003; Sirzen-Zelenskaya et al., 2006; Hamad et al., 2011, 2014, 2021, 2024; Rajan et al., 2021). Moreover, the microbiome exerts a distinct influence on dendritic structure, either by preserving or altering it. In line with previous findings, the administration of galacto-oligosaccharides (GOS), a well-studied prebiotic, for 40 days has been shown to increase dendritic spine density in rats, which is a reliable indicator of hippocampal excitatory synapses (Waworuntu et al., 2016). In contrast, GF mice exhibited aberrant mushroom-shaped dendrites that were thinner, shorter, and shrunken compared with control mice. This reduced synaptic strength and plasticity, despite an increase in dendritic branching (Ferrante et al., 2013). Additionally, the mode of delivery affects the composition of the microbiota, which may influence dendritic arborization. Studies have shown that mice and rats delivered by caesarean section (C-section) have exhibited decreased dendritic arborization or branching (Juárez et al., 2008; Chiesa et al., 2019). The transplantation of microbiota from aged experimental animals to young recipients has been observed to result in a reduction in dendritic spines in the hippocampus and PFC as well as impaired memory performance and altered neuron plasticity protein expression (D’Amato et al., 2020; Li et al., 2020). The collective evidence indicates that the gut-brain axis can influence the morphology of distinct types of neurons. Consequently, supplementation of a mouse model of amyotrophic lateral sclerosis (ALS) with GOS for 74 days has resulted in an increased abundance of *Bifidobacterium* and *Lactobacillus* genus, thereby reducing motor neuron death and spinal cord inflammation when compared with the untreated group (Song et al., 2013). In contrast, GF mice had pyramidal neuron atrophy, and a reduction in the branching of these cells in the hippocampal DG and amygdala (London et al., 2013).

#### 2.1.4 Microglia morphology

Microglia regulate synaptic plasticity, and phagocytosis, promoting the survival of neurons and neural progenitors by releasing growth factors that maintain neurons’ homeostasis and function (Tay et al., 2017). The interactions between the microbiota, the immune system, and the brain are currently being recognized as crucial mechanisms that shape the maturation, activation, and morphology of microglia (Rea et al., 2016; Vuong et al., 2017). This hypothesis is supported by the observation that GF mice exhibit immature microglia in the corpus callosum, cortex, hippocampus, olfactory bulb, and cerebellum, accompanied by an abnormal density and morphology. However, these abnormalities were reversed by microbiota colonization with SCFAs (Erny et al., 2015). In contrast, a stroke model of GF mice exhibited a reduction in the number of microglia (Singh et al., 2018). The administration of an antibiotic cocktail to C57BL/6 mice resulted in the induction of gut dysbiosis, which was accompanied by abnormal activation of microglia and astrocytes, indicative of inflammation in the hippocampus (Guida et al., 2018). Moreover, the induction of gut perturbations in the mouse model of Parkinson’s disease (PD) resulted in a reduction of microglial diameter and overall size in the caudate-putamen and substantia nigra, thereby impairing their function (Sampson et al., 2016). It is evident that Bacteria species such as *Lactobacillus helveticus*, which is known as *Lactobacillus acidophilus* in natural human inhabitants, as well as *Bifidobacterium longum*, can regulate microglia and synaptogenesis in the hypothalamus in response to induced stress (Ait-Belgnaoui et al., 2014). *Lactobacillus delbrueckii*, *Lactobacillus casei*, *Lactobacillus acidophilus*, and exogenous *Lactobacillus plantarum* have been demonstrated to regulate microglial activation in aged Wistar rats’ hippocampus (Distrutti et al., 2014). It has been demonstrated that in an obese-insulin-resistant rat model, *Lactobacillus casei* supplementation for 12 weeks was able to restore microglia function by regulating their activation (Chunchai et al., 2018). The use of antibiotics was found to result in a reduction in the process of microglia-mediated synapse engulfment, which is associated with a reduction in synapse density observed in postmortem cortical tissue of individuals with schizophrenia (SCZ) (Sellgren et al., 2019).These findings indicate that a healthy and diverse GI microbiome is essential for maintaining healthy microglia and optimal cognitive function (Cryan and Dinan, 2015; Thion et al., 2018).

#### 2.1.5 BBB structure and permeability

The BBB is a complex structure comprising endothelial cells, which are held together by tight junctions and adherens junctions, and interact with pericytes, the basement membrane of capillaries, microglia, and astrocytes (Ary et al., 2023). The primary function of the BBB is to safeguard the brain from toxic substances, facilitate their removal from the brain to the bloodstream, and provide essential nutrients to brain tissue. Consequently, the BBB is subject to rigorous regulation, and most neurological disorders are characterized by elevated permeability. A growing body of evidence from clinical and experimental studies supports the role of the gut-brain axis in regulating the integrity of BBB (Esposito et al., 2002; Braniste et al., 2014; Montagne et al., 2015; Thevaranjan et al., 2017; Dhaliwal et al., 2018; Li et al., 2018). Mice with normal microbiota develop a BBB around postnatal day 14. At postnatal day 15, the permeability of the BBB begins to decrease, while GF mice exhibit increased BBB permeability and decreased endothelial tight junction proteins at postnatal day 16. This suggests that gut bacteria play a role in maintaining the integrity of the BBB, as colonization with bacteria restores its function and normal selectivity (Braniste et al., 2014). *Lactobacillus plantarum*, a Gram-positive lactic acid bacterium that is frequently present in fermented food products, has been demonstrated to increase the integrity of the BBB in Swiss albino mice when supplemented for 28 days in rats (Dhaliwal et al., 2018). Moreover, administration of *Clostridium butyricum* 14 days prior to and following traumatic brain injury in mice has been demonstrated to mitigate neuronal degeneration and BBB permeability (Li et al., 2018). Clinical studies have indicated that both age and stress can compromise the integrity and function of the GI barrier because of microbial dysbiosis, which in turn impacts the permeability of the BBB, thereby hastening the process of inflammation associated with aging. These findings are corroborated by the observation that bacterial metabolites, such as butyrate and propionate, enhance the integrity of the epithelial barrier by facilitating the assembly of tight junctions (Peng et al., 2009; Tong et al., 2016).

#### 2.1.6 Synapses structure and function

Synapses are the points of contact between neurons, where connections are established, and signals are transmitted between them. The number of synaptic connections possessed by neurons may vary, ranging from a few to hundreds of thousands. These connections may link to the neuron itself, adjacent neurons, or neurons in different brain areas (Caire et al., 2021). Synaptic plasticity is evaluated by the capability of neurons to adjust the intensity of their connections, which plays a crucial role in the formation and reconstruction of brain networks following disruption (Stampanoni Bassi et al., 2019). A measure of synaptic plasticity that controls the strength of neuro-connectivity is long-term potentiation (LTP) and long-term depression. These are persistent alterations in synaptic strength (Bliss and Cooke, 2011) which have been demonstrated to be influenced by gut microbes (Maren and Quirk, 2004). Synaptic plasticity in the hippocampus is primarily regulated by glucocorticoids, which are metabolized by microbiome bacteria such as *Eggerthella lenta*, *[Clostridium] scindens 1*, and *[Clostridium] scindens 2*. These bacteria influence changes in synaptic function and excess neuronal injury (Lupien et al., 2005; Chen et al., 2006; Ridlon et al., 2013; Morris and Ridlon, 2017), which were not observed in GF mice (Bokkenheuser et al., 1984). Another study of GF mice in a model of Huntington’s disease demonstrated alterations in plasticity (Radulescu et al., 2019). Moreover, *Lactobacillus casei* has been linked to the regulation of synaptogenesis, synaptic refinement, and pruning in the hippocampus, which is crucial in the context of brain disorders involving neurodegeneration (Ogbonnaya et al., 2015; Guida et al., 2018; Sawada et al., 2018). A novel probiotic composition, comprising bifidobacteria (*Bifidobacterium longum*, *Bifidobacterium breve*, *Bifidobacterium infantis*), lactobacilli (*Lactobacillus acidophilus*, *Lactobacillus plantarum*, *Lactobacillus casei*, *Lactobacillus delbrueckii subsp. bulgaricus*), and *Streptococcus thermophilus*, has been demonstrated to enhance neuroplasticity in a mouse model of Alzheimer’s disease (AD), effectively reversing deficits in LTP (Bonfili et al., 2017). Another study has demonstrated the pivotal role of *Lactobacillus reuteri* in rectifying deficits in synaptic plasticity, which resulted in the amelioration of social behaviors (Zhu et al., 2017). The combination of a probiotic with a prebiotic (synbiotic), including *Lactobacillus casei* with inulin, was found to significantly mitigate synaptic plasticity and increase 5-HT1A in the hippocampus region, particularly in the CA1 and DG of healthy juvenile rats (Barrera-Bugueño et al., 2017). Despite the adversities in quality control and manufacturing of synbiotics, they have been used in microbiome-based therapies for AD, which influenced desirable insulin regulation and amyloid plaque reduction (Nakhal et al., 2024). The findings highlight the importance of the connections between the MGBA and brain morphology, as outlined in Table 1.

**Table 1:** The relationships between the MGBA and brain morphology.

Aspect	Key findings	Microbiota involved	References
Neurogenesis	*Lactobacillus casei* ↑ DG neurogenesis, synaptic activity, BDNF–TrkB signaling. Butyrate-producing bacteria ↑ hippocampal neurogenesis.	*Lactobacillus casei, Faecalibacterium prausnitzii, Anaerobutyricum hallii, [Eubacterium] rectale, Coprococcus* spp.*, [Butyricicoccus] pullicaecorum, Clostridium butyricum*	Möhle et al. (2016), Guida et al. (2018), Sawada et al. (2018), and Fusco et al. (2023)
Myelination	Dysbiosis and *Akkermansia* spp. ↓ myelin integrity. Butyrate ↑ regulation of myelination gene expression.	*Akkermansia muciniphila, Clostridium butyricum, butyrate-producing bacteria*	Hoban et al. (2016), Hughes and Appel (2016), and Cekanaviciute et al. (2017)
Dendritic morphology	Prebiotics ↑ dendritic spine density. Aged microbiota transfer ↓ dendritic spines & memory performance.	*Bifidobacterium* spp.*, Lactobacillus* spp.*, GOS (prebiotic)*	Waworuntu et al. (2016)
Microglia morphology	Dysbiosis induces microglial abnormalities Probiotics ↑ microglial function in aged or diseased models	*Lactobacillus helveticus, Bifidobacterium longum, SCFA-producing bacteria*	Ait-Belgnaoui et al. (2014), Rea et al. (2016), and Vuong et al. (2017)
BBB integrity	Probiotics ↑ BBB integrity SCFAs ↑ tight junction assembly	*Lactobacillus plantarum, Clostridium butyricum, SCFA-producing bacteria*	Peng et al. (2009), Tong et al. (2016), and Dhaliwal et al. (2018)
Synapses	GF mice → altered synaptic plasticity, reduced connectivity Probiotics and synbiotics ↑ synaptogenesis	*Lactobacillus casei, Bifidobacterium* spp.*, Streptococcus thermophilus, synbiotics (probiotic + prebiotic combinations)*	Möhle et al. (2016) and Sawada et al. (2018)

Germ-free (GF), Dentate gyrus (DG), Brain-derived neurotrophic factor-tropomyosin receptor kinase B (BDNF–TrkB), Blood–brain barrier (BBB), Oligosaccharides (GOS), and Short-chain fatty acids (SCFAs).

## 3 The MGBA and neuroplasticity

**Figure 2:**
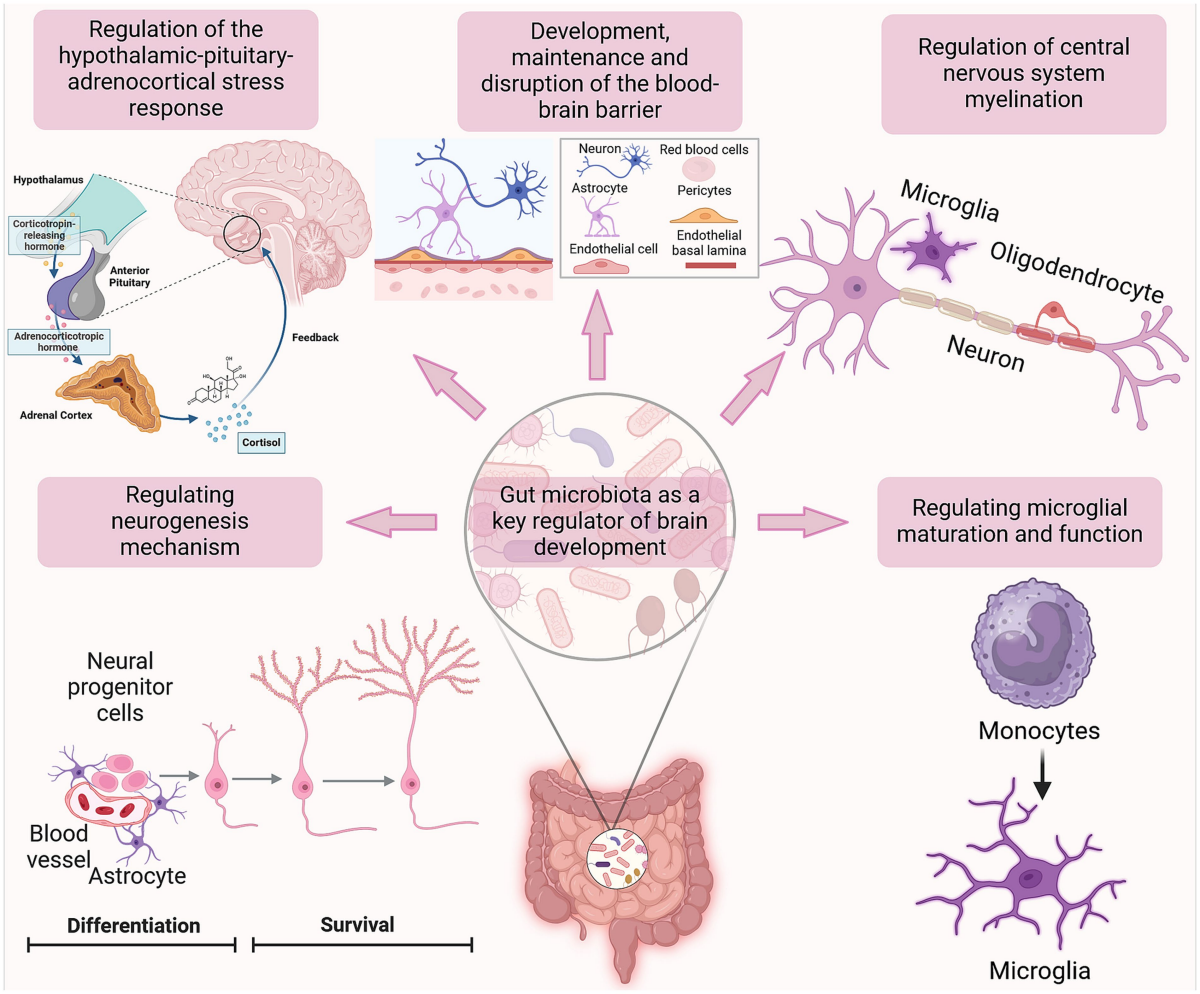
The impact of gut microbiota on brain development. The figure illustrates the correlation between alterations in gut microbiota and hippocampal neurogenesis, as well as the association between these changes and the development of neurological disorders. The gut microbiota plays a crucial role in brain development, including neuroplasticity, by regulating neurogenesis, microglial maturation, the HPA axis, and CNS myelination. These processes may be influenced by the gut microbiota, either positively or negatively, resulting in the promotion or disruption of their development. Created with BioRender.com.

The accumulating evidence linking bacteria in the gut and neurons in the brain has prompted a paradigm shift in neurosciences. Neuroplasticity is the brain’s ability to reorganize itself through the formation of new neural connections and the construction of novel networks in response to learning, experience, or injury. This process, known as neurogenesis, is a fundamental aspect of brain development. This phenomenon allows the brain to adapt and change throughout life, influencing various behaviours. Conversely, the gut microbiota comprises a varied population of microorganisms inhabiting the digestive system, which is pivotal for digestion, and immune response. The link between neuroplasticity and the gut microbiota stems from the microbiota’s ability to affect brain function and behaviour via multiple pathways, including neurotransmitter production, immune system modulation, and inflammation regulation. Consequently, this interplay may influence both cognitive functions and mental health. Maintaining a balanced gut microbiota is crucial for proper gut physiology and signaling of the MGBA (Figure 2). When there is an imbalance in the microbiota or its functions, known as dysbiosis, it can have adverse effects on various systems, including the GI tract and the CNS. Thus, the understanding of the mechanisms of synaptic plasticity may provide essential insights into the pathophysiological nature of neuropsychiatric disorders, such as Attention-deficit/hyperactivity disorder (ADHD), and neurological disorders, including epileptic seizures, pointing to new therapeutic interventions.

### 3.1 Diverse mechanisms of neuroplastic adaptation

Plasticity involves a variety of mechanisms, including synaptic plasticity, neurogenesis, and changes in glial cell function, which enable neurons to form new connections, strengthen existing ones, and weaken others (Dzyubenko and Hermann, 2023). One research group demonstrated that the calcium-binding protein S100B in the cytoplasm and nucleus of astrocytes significantly modulates long-term synaptic plasticity (Nishiyama et al., 2002). Astrocytes release glutamate through a natural increase in their internal calcium levels, subsequently inducing significant glutamatergic activity in nearby neurons (Parpura and Haydon, 2000). Oligodendrocyte precursor cells receive glutamatergic signals directly from hippocampal pyramidal neurons (Bergles et al., 2000). These findings suggest that bidirectional communication between glial cells and neurons may contribute to synaptic plasticity. Another study showed that mice lacking S100B mutants grew normally and had no observable abnormalities in brain cytoarchitecture. However, these mutants displayed enhanced synaptic plasticity, as evidenced by increased LTP in the hippocampal CA1 region. Remarkably, it’s evident that a glial protein has been shown to modulate neuronal synaptic plasticity, working memory, and learning (Nishiyama et al., 2002). This offers a hallmark feature for the understanding of numerous diseases, including the neurodegenerative ones, and their association with the dysbiosis induced by astrocytic dysfunction. In terms of cognitive aspects, neural plasticity can be defined as the ability to modify the functioning of neural circuits based on experience, thus influencing thoughts, feelings, and behavior (Citri and Malenka, 2008). The neuroplasticity that involves changes in the strength and number of synapses between neurons over time is called synaptic plasticity.

There are two main forms of synaptic plasticity: Hebbian and homeostatic plasticity (Galanis and Vlachos, 2020). Hebbian plasticity involves a change in synaptic strength mediated by increasing or decreasing neuronal activity after the onset of stimulation and is involved in lifelong changes (Vitureira and Goda, 2013). It plays a critical role in learning and memory. In 1966, Terje Lømo and Tim Bliss studied the effects of activating the perforant path to dentate granule cells in the hippocampus of anesthetized rabbits, and they observed that brief trains of stimuli resulted in increased efficiency of transmission at the perforant path-granule cell synapses that could last for hours. When they applied a burst of tetanic stimulus to the perforant path fibers, it resulted in a dramatic and long-lasting enhancement in the post-synaptic response of cells in the dentate gyrus. In 1968, both scientists proposed the role of the hippocampus in certain forms of memory (Lømo, 2003).

Homeostatic plasticity constitutes a negative feedback loop in response to increased neuronal activity. It involves the regulation of neuronal excitability or the stabilization of overall synaptic strength (von Bernhardi et al., 2017). Also, it involves coordinated changes among distinct parts of the neuron, such as the synapses, the cell body, and the axon. White matter plasticity or myelin plasticity offers a way in which the structure of white matter can be altered by experience (Sampaio-Baptista et al., 2020). Despite the importance of myelination in circuit activity, there remains a lack of understanding of how neuronal activity and brain plasticity might impact myelination. Myelin, which is formed and wrapped around axons by oligodendrocytes, originates from embryonic neural progenitors, progressing through stages of OL precursor or progenitor cells before maturing into oligodendrocytes. Apart from direct alterations to existing myelin sheaths, an essential mechanism regulating myelin plasticity involves the genesis, differentiation, and proliferation of myelinating glia (Chorghay et al., 2018). Receptors located in axons and cells originating from the oligodendrocytes lineage detect neuronal activity. These cells utilize the metabolites derived from this activity to either maintain the myelin sheaths, such as through lipid synthesis, or to offer metabolic support by transporting lactate (Chorghay et al., 2018). As myelination progresses rapidly in early life, perturbations in the neonatal gut microbiota during initial colonization could disrupt proper myelination by insidiously affecting the immune responses and neuronal differentiation. The effects of neonatal antibiotic-induced dysbiosis on the development of the MGBA, including aspects such as myelination and behaviour, remain unclear. Neonatal antibiotic-induced dysbiosis affects myelination and behaviour in mice, and it was hypothesized that neonatal antibiotic-induced dysbiosis disrupts host–microbe interactions, resulting in impaired myelination in the brain and changes to the MGBA (Keogh et al., 2021). The introduction of the SCFA butyrate helped reverse myelination deficits in the PFC. The presence or absence of certain bacterial taxa, such as *Bacteroidaceae* and *Coriobacteriaceae*, is known to be important for short-chain fatty acid production and is reduced in certain conditions like MS, suggesting an indirect link between gut dysbiosis and altered myelination (Yan and Charles, 2018). The study highlighted a persistent effect of neonatal antibiotic treatment on the MGBA, impacting myelin regulation in the PFC, and reducing cognitive function. Furthermore, bacterial metabolites were found to effectively reverse this altered phenotype, and the gut microbiota showed a critical role in mediating these effects (Keogh et al., 2021).

### 3.2 Brain plasticity modulation by gut microbiota through neurotrophic factors

**Figure 3:**
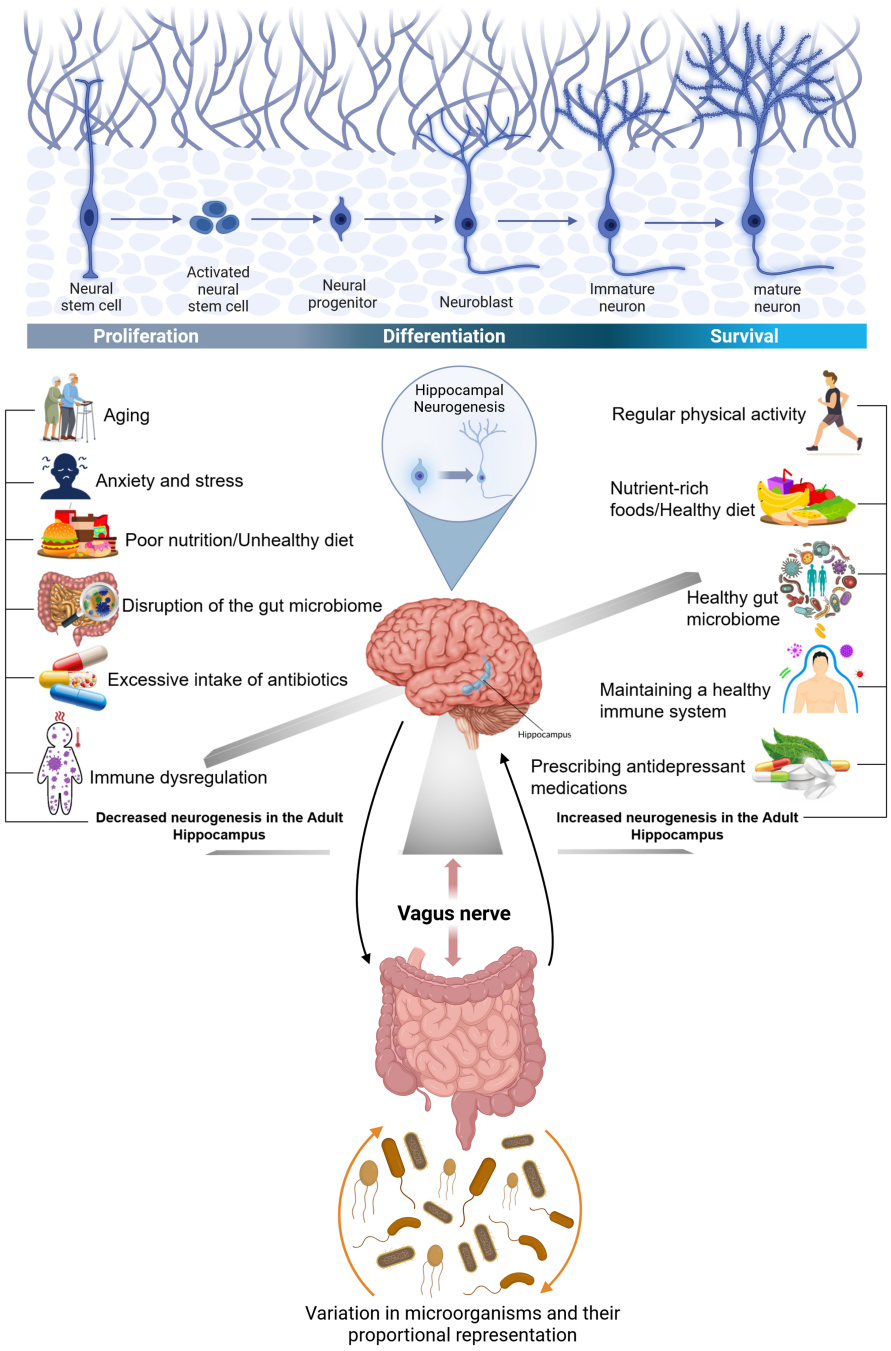
The connection between gut bacteria and the brain and factors influencing hippocampal neurogenesis. The GI tract is connected to the brain through an important nerve called the vagus nerve. The vagus nerve originates in the brain stem and extends through the neck, chest, and abdomen. In the human body, the nervous system and another axis and the HPA axis are the main systems that respond to stress. Prolonged stress can adversely affect gut bacteria due to the interconnectedness of the HPA axis with the gut-brain axis: Furthermore, alterations in the gut microbiota can also influence the brain, potentially increasing susceptibility to brain conditions like depression. Regular physical activity regularly, a nutrient-rich diet, and the prescription of antidepressants are key factors that have a direct impact on our functional microbiota-gut-brain axis, resulting in a healthy immune system and an increased neurogenesis in the adult hippocampus. However, following an unhealthy diet with a high concentration in saturated fat, overuse of antibiotics, and being under stress are other markers that abnormally influence the physiological processes of the immune and endocrine systems, resulting in a decreased neurogenesis in the adult hippocampus, and disruption of the gut microbiome. The human gut is composed of dominant phyla, Bacteroidetes, and Firmicutes, and the activity and electrical properties of enteric neurons can be influenced by gut bacteria through actions on ion channels, thus affecting their adaptability. Created with BioRender.com.

BDNF plays various roles in brain physiology. It is involved in LTP and synaptic plasticity, where it shapes the morphology of mature neurons by promoting axonal outgrowth and pruning which is a natural process that removes excess or unused synapses, allowing the brain to optimize its efficiency and function. The gut microbiota can control the expression of a variety of neurotrophic factors, such as BDNF and glial cell line-derived neurotrophic factor. The glial cell line-derived neurotrophic factor was initially thought to be able to regulate the growth, survival, and differentiation of neural-derived cell types. However, these factors and their receptors are also widely found to be expressed across many different neurodegenerative diseases, like AD, and PD (Azman and Zakaria, 2022), epilepsy (Leifeld et al., 2022) as well as psychiatric disorders, such as major depressive disorder (MDD), and autism spectrum disorder (ASD). Several communication pathways connecting the gut microbiota and the brain have been delineated. These include neural pathways through the vagus nerve and the ENS, immune signaling mediated by cytokines due to the high concentration of immune cells in the gut, and hormonal modulation via the HPA axis, leading to changes in glucocorticoid levels. The vagus nerve relays vital information between the brain and the GI, respiratory, and cardiovascular systems (Figure 3).

### 3.3 Gut microbiota and aging

The impact of the microbiota on brain plasticity in the elderly extends to exploring its potential implications for neurological health and age-related cognitive decline. One study found that GF mice exhibited decreased levels of BDNF expression in both the cortex and hippocampus, as well as reduced expression of the NR2B subunit of the N-methyl-D-aspartate (NMDA) receptor. Moreover, NMDA receptors contribute to memory formation by modulating synaptic plasticity (Li and Tsien, 2009). Furthermore, there is a decrease in adult hippocampal neurogenesis in the elderly, which parallels a decline in cognitive function and synaptic plasticity. Therefore, it could be inferred that the pathophysiological effects of microbiota are not recognized until old age. More specifically, as individuals age, there are physiological changes that affect metabolic, genomic, and immunological functions, leading to an increased susceptibility to infections and diseases. These changes can result in a condition known as inflammaging, which is a chronic, low-grade inflammation associated with age-related diseases.

Witte et al. (2009) conducted the initial study showing the beneficial effects of calorie restriction on memory performance in an elderly group. Similarly, intermittent fasting in mice led to increased markers of brain plasticity (Singh et al., 2012). Exploring the complex relationship between gut microbiota and synaptic plasticity in the elderly offers the potential to develop innovative therapeutic approaches to mitigate age-related cognitive decline.

## 4 Method for studying the MGBA

There are a variety of tools and techniques which has been recently introduced to study the MGBA, paving the way for researchers to narrow the gaps in understanding of the MGBA. These include the GF mouse model, antibiotic-induced dysbiosis, FMT, probiotics, and prebiotics. In this chapter, we will focus on the GF mice as a tool to study the MGBA.

### 4.1 The GF mice

The GF mice, which have been exposed to no microbial agents, serve as a model for investigating the intricate interplay between the gut microbiota and the gut-brain axis. Microbiological evaluation of these mice reveals the complete lack of growth for anaerobic, aerobic, and mycotic bacteria, confirming their GF status. Studies utilizing GF mice have yielded compelling evidence for the involvement of gut microbiota in the gut-brain axis.

### 4.2 Studying brain morphology using the GF mice

#### 4.2.1 Studying microglia using the GF mice

Several studies have demonstrated that GF mice, exhibit differences in microglial gene expression and morphology compared to mice with normal gut bacteria (Abdel-Haq et al., 2019; Huang et al., 2023). In contrast to mice with normal gut bacteria, GF mice have a greater number of microglia in their brains. However, these cells exhibit abnormal shapes and a reduced ability to respond to infection (Erny et al., 2015; Zhang et al., 2022). Similarly, antibiotic treatment in normal mice also results in analogous microglial alterations, thereby indicating that gut bacteria are of pivotal importance for microglial development and functionality (Çalışkan et al., 2022). The reintroduction of a complex mixture of gut bacteria or SCFAs, produced by gut bacteria, was found to restore normal microglial function in GF mice (Erny et al., 2015). Moreover, a recent study demonstrated that the gut microbiota exerts a significant influence on age-related changes in microglial function. By comparing microglial gene expression in young-adult and aged mice under GF and specific pathogen-free conditions, the authors reported that the absence of gut microbiota in GF mice reduced oxidative stress and improved mitochondrial function in aged microglia (Mossad et al., 2022). Moreover, they also reported that metabolomic analyses revealed that N6-carboxymethyllysine accumulated in aging brains of specific pathogen-free mice, impairing mitochondrial activity and increasing oxidative stress. This age-related increase in N6-carboxymethyllysine was validated in both mice and human samples and linked to microbiota-induced intestinal permeability, thus highlighting the gut-brain axis’s role in microglial aging (Mossad et al., 2022).

#### 4.2.2 Studying astrocytes using the GF mice

Astrocytes, the primary glial cells in the brain, play a central role in a multitude of metabolic interactions with neurons, regulation of blood flow, homeostasis of extracellular fluid, ions, and transmitters, energy provision, regulation of synapse function, and synaptic remodelling (Gradisnik and Velnar, 2023). Astrocytes provide fuel for neuronal activity by releasing lactate in response to synaptic glutamate release. This process is designated as the astrocyte-neuron lactate shuttle. Recent research has identified a critical influence of the gut microbiota on the modulation of genes involved in the astrocyte-neuron lactate shuttle, a key component of brain energy metabolism. In a 2020 study, Margineanu and colleagues demonstrated that microbial colonization of GF mice led to the upregulation of Atp1a2 (encoding the ATPase, Na+/K+ transporting, alpha 2 sub-unit) and Pfkfb3 (encoding 6-phosphofructo-2-kinase/fructose-2,6-biphosphatase 3), which is predominantly expressed in astrocytes and is crucial for their metabolic functions (Margineanu et al., 2020). ATP1A2 is essential for maintaining ionic balance and membrane potential in astrocytes, while PFKFB3 plays a key role in glycolysis and lactate production. Specifically, the mRNA levels of Atp1a2 and Pfkfb3 were elevated in the hippocampus of mice colonized with microbes for 24 h, in comparison to conventionally raised mice (Margineanu et al., 2020). Furthermore, Pfkfb3 was upregulated in GF mice, while the increase in Atp1a2 was confirmed at the protein level by Western blot analysis. Furthermore, in an animal model of chronic psychosocial stress, 6-week dietary supplementation with prebiotics (fructo-and galacto-oligosaccharides) also upregulated Atp1a2 and Pfkfb3 mRNA expression in the hippocampus (Margineanu et al., 2020). These findings indicate that the gut microbiota and prebiotics can significantly influence the metabolic coupling between neurons and astrocytes by modulating the expression of key genes, including Atp1a2, Pfkfb3, lactate dehydrogenase A, lactate dehydrogenase B, monocarboxylate transporter 1, and glycogen synthase 1.

#### 4.2.3 Studying BBB using the GF mice

A study by Gong et al. (2024) investigated the impact of gut microbiota dysbiosis on BBB integrity using GF mice that underwent FMT from patients with anti-(NMDA) receptor encephalitis. The dysbiotic microbiota exhibited a low short-chain fatty acid content and an altered bacterial composition, including a decrease in *Lachnospiraceae* and an increase in *Verrucomicrobiota*, *Akkermansia*, *Parabacteroides*, and *Oscillospirales* (Gong et al., 2024). Following FMT, the mice exhibited increased susceptibility to an encephalitis-like phenotype, characterized by behavioural deficits, and elevated T2-weighted image hyperintensities (Gong et al., 2024). The study demonstrated that dysbiosis resulted in impaired BBB integrity, as evidenced by increased Evan’s blue dye extravasation and reduced expression of tight junction proteins ZO-1 and claudin-5. Furthermore, proinflammatory cytokines (IL-1, IL-6, IL-17, TNF-*α*, and LPS) were elevated, contributing to a pro-inflammatory state and significant brain inflammation, particularly in the hippocampus and cortex, with decreased NMDA receptor expression (Gong et al., 2024). These findings underscore the crucial role of the gut microbiota in maintaining the integrity of the BBB and suggest that dysbiosis may exacerbate susceptibility to neuroinflammatory conditions such as NMDAR-excitotoxicity.

A further study conducted in 2022 on GF mice demonstrated that the absence of gut microbiota leads to increased BBB permeability. This is evidenced by a reduction in the expression of tight junction proteins, including claudin-5 and occludin, which are essential for maintaining the integrity of the BBB (Park and Im, 2022). Consequently, GF mice exhibit greater cognitive disabilities than mice with a normal microbiota. Moreover, the administration of specific probiotics and microbiota-derived metabolites, such as SCFAs, has been demonstrated to protect and enhance BBB integrity (Park and Im, 2022). This underscores the pivotal role of the gut microbiota in regulating BBB permeability and safeguarding against neuroinflammation and cognitive impairments.

**Figure 4:**
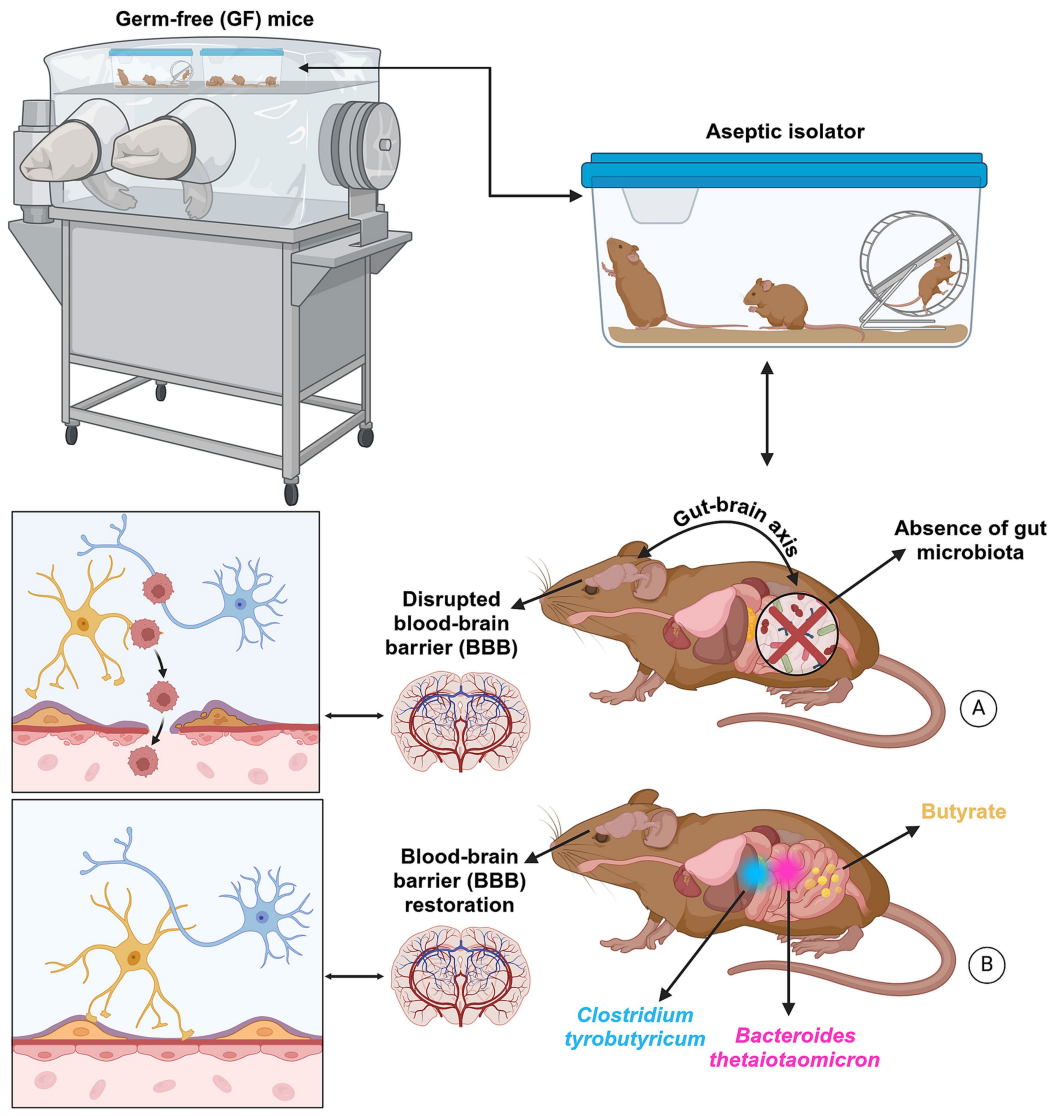
The BBB is not fully developed in GF mice. This barrier controls the biological substances essential for brain metabolic activity and neuronal function. Thus, the functional and structural integrity of the BBB is critical for preserving molecules circulating in the blood system and maintaining homeostasis of the brain microenvironment. When the BBB is compromised, it negatively impacts neurogenesis during early development by exposing the brain to excessive glucocorticoid molecules **(A)**. Consequently, cognitive deficits and neuropsychological symptoms are highly prevalent during the long-term effects of glucocorticoids. However, a large body of evidence suggests that gut microorganisms may regulate cell junctions in the blood–brain barrier structure. GF mice treated with either *Clostridium tyrobutyricum*, a rod-shaped Gram-positive bacterium that grows under anaerobic conditions, produces butyric acid, decreases the expression of pro-inflammatory cytokines, and increases the diversity of the gut microbiota, or *Bacteroides thetaiotaomicron*, which processes various polysaccharides and interacts with other gut microbes, showed an improvement in the integrity of the BBB **(B)**. Identical effects were observed microscopically when GF mice were directly treated with butyrate, which enhances intestinal barrier function and mucosal immunity, highlighting its important impact on the MGBA. Created with BioRender.com.

When the BBB is compromised, it negatively impacts neurogenesis during early development by exposing the brain to excessive glucocorticoid molecules (Figure 4A). Consequently, cognitive deficits and neuropsychological symptoms are highly prevalent during the long-term effects of glucocorticoids. However, a large body of evidence suggests that gut microorganisms may regulate cell junctions in the BBB structure. GF mice treated with either *Clostridium tyrobutyricum* (Braniste et al., 2014), a rod-shaped Gram-positive bacterium that grows under anaerobic conditions, produces butyric acid, decreases the expression of pro-inflammatory cytokines, and increases the diversity of the gut microbiota, or *Bacteroides thetaiotaomicron*, which processes various polysaccharides and interacts with other gut microbes (Durant et al., 2020; Rutsch et al., 2020), showed an improvement in the integrity of the BBB (Figure 4B). Identical effects were observed microscopically when GF mice were directly treated with butyrate, which enhances intestinal barrier function and mucosal immunity.

#### 4.2.4 Studying neurogenesis using the GF mice

A growing body of evidence indicates that neurogenesis in the hippocampus, is influenced by the gut microbiota. In GF mice, the survival of newly generated neurons in the subgranular zone of the hippocampus is increased, which is associated with spatial memory. Nevertheless, cell proliferation remains unaltered (Guzzetta et al., 2022). This enhancement in neurogenesis may be linked to the observed deficits in spatial learning and memory in GF mice. More recent studies have highlighted the significant impact of the gut microbiota on neurogenesis. Kundu et al. (2019) further expanded upon these findings by demonstrating that young GF mice that received gut microbiota transplants from older mice exhibited increased hippocampal neurogenesis and intestinal growth. The transplanted microbiota led to an enrichment of butyrate-producing microbes, resulting in elevated levels of fibroblast growth factor 21 (Kundu et al., 2019). This increase in fibroblast growth factor 21 correlated with enhanced activation of AMPK and SIRT-1, along with reduced mTOR signaling, suggesting a mechanistic link between gut-derived metabolites and brain health (Kundu et al., 2019). The findings indicate the significance of the microbiota in brain morphology using GF mice, which are summarized in Table 2.

**Table 2:** Specific brain structures studied in GF mice.

Condition		Gut microbiota status	Observed effect	Genes/mechanisms	Reference
Brain morphology	Microglia	GF mice	↑ Microglia numbers, abnormal shapes, reduced infection response, changes in gene expression	Microglial genes, SCFAs	Luczynski et al. (2016)
		GF mice	↓ Oxidative stress in aged microglia; improved mitochondrial function	CML, oxidative stress genes	Mossad et al. (2022)
	Astrocytes	GF mice	↑ Atp1a2 and Pfkfb3 mRNA in hippocampus, upregulated Atp1a2 protein levels, ↑ metabolic coupling genes	Atp1a2, Pfkfb3, LDHA, LDHB, Mct1, Gys1	Margineanu et al. (2020)
	BBB	GF with FMT from NMDARE patients	↑ BBB permeability; ↓ ZO-1, claudin-5 expression; ↑ proinflammatory cytokines	Claudin-5, proinflammatory cytokines (IL-1, IL-6, IL-17, TNF-α, LPS)	Gong et al. (2024)
		GF mice	↑ BBB permeability; ↓ tight junction proteins expression	Claudin-5, occludin	Park and Im (2022)
	Neurogenesis	GF mice	↑ Survival of newly generated neurons in SGZ; spatial memory deficits	Not specified	Luczynski et al. (2016)
		GF with older microbiota	↑ Hippocampal neurogenesis; ↑ intestinal growth; ↑ FGF21	AMPK, SIRT-1, mTOR, FGF21	Kundu et al. (2019)

Short-chain fatty acids (SCFAs), N6-carboxymethyllysine (CML), ATPase Na+/K+ Transporting Subunit Alpha 2 (ATP1A2), 6-phosphofructo-2-kinase/fructose-2,6-biphosphatase 3 (PFKFB3), lactate dehydrogenase-A or B (LDHA, LDHB), monocarboxylate transporter 1 (MCT1), glycogen synthase 1 (GYS1), Zonula Occludens-1 (ZO-1), blood–brain barrier (BBB), Anti-N-Methyl-D-aspartate Receptor Encephalitis (NMDARE), Germ-free (GF), fecal microbiota transplantation (FMT), subgranular zone (SGZ), Fibroblast growth factor-21 (FGF21), Interleukin (IL), Tumor necrosis factor alpha (TNF-α), Adenosine monophosphate-activated protein kinase (AMPK), Sirtuin 1 (SIRT-1), Mammalian target of rapamycin (mTOR), and Lipopolysaccharide (LPS).

### 4.3 Neuroimmune and neuroinflammatory disorders using the GF model

#### 4.3.1 Studying PD using the GF mice

Recent studies have demonstrated that the gut microbiota can influence brain function and behaviour, with the potential to contribute to neurodegenerative diseases, such as PD. The degeneration of dopaminergic neurons is a hallmark of PD, and there is evidence that changes in the composition of the gut microbiota can lead to alterations in the brain’s dopaminergic systems. A recent study has demonstrated that GF mice exhibited significant dopaminergic dysfunction in comparison to specific pathogen-free mice, suggesting an increased susceptibility to PD (Wang et al., 2023). The GF mice exhibited lower dopamine levels in the frontal cortex, striatum, and hippocampus, as well as altered tyrosine hydroxylase (TH) expression, with decreased TH mRNA in the cerebellum and reduced TH protein in the striatum (Wang et al., 2023). This highlights the critical role of gut microbiota in maintaining dopaminergic function and its involvement in PD pathology.

#### 4.3.2 Studying ASD using the GF model

In a study conducted by Berding and Donovan (2019), researchers examined the effects of gut microbiota on ASD by transplanting fecal microbiota from children with ASD into GF mice. The study revealed that the recipient mice exhibited behavioural changes and GI alterations analogous to those observed in individuals with ASD, including increased anxiety-like behaviors and disrupted gut function (Berding and Donovan, 2019). These changes were associated with alterations in the expression of several genes involved in immune function and neural signalling, including those related to synaptic plasticity and inflammation. Notably, the ASD microbiota promoted extensive alternative splicing of ASD-relevant genes in the brain, particularly affecting genes such as Cntnap2, Shank3, and Gabrb3, which are implicated in ASD pathology (Berding and Donovan, 2019). In contrast, other studies claimed that the absence of microbiota, results in various ASD like behaviors and neurodevelopmental changes, such as increased anxiety, reduced sociability, and neurodevelopmental alterations, that mimics ASD symptoms (Desbonnet et al., 2014; Arentsen et al., 2015). The specific bacterial taxa involved included a reduction in the levels of *Bifidobacterium* and an increase in the levels of *Lactobacillus* and *Desulfovibrio.* Although the transplanted microbiota did not result in the development of autism in the mice, the findings indicate that the composition of the gut microbiota plays a pivotal role in influencing ASD-related symptoms. This underscores the potential for gut microbiota-targeted therapies in managing ASD symptoms.

#### 4.3.3 Studying Crohn’s disease (CD) using the GF mice

A recent study revealed that the transplantation of microbiota from patients with CD to GF mice resulted in the development of colitis (Sheikh et al., 2024). To investigate the potential association between CD and the gut microbiota in the context of the gut-brain axis, researchers collected fecal samples from healthy controls and CD patients. GF mice were inoculated with these samples, resulting in distinct transcriptomic profiles in the colon. GF mice with CD microbiota exhibited increased susceptibility to Crohn’s-like symptoms, as evidenced by histopathology and immunohistochemistry (Sheikh et al., 2024). Principal component analysis revealed distinct clustering, with inflammatory pathways such as chemokine signalling and leukocyte transmigration significantly enriched. The expression of CD74, TNF, and cytokine receptors was found to be elevated in GF mice with CD microbiota, indicating an augmented immune response. The GF mice exhibited a greater susceptibility to CD compared to those with a healthy microbiota, as evidenced by significant inflammation and immune cell infiltration (Sheikh et al., 2024). This suggests that the gut microbiota plays a significant role in the pathogenesis of CD.

#### 4.3.4 Studying anxiety using the GF mice

In their 2021 study, Kamimura and colleagues investigated the behavioural and neurobiological consequences of microbiota deficiency in GF mice. The results indicated that GF mice exhibited increased anxiety-like behaviours compared to specific pathogen-free mice, as evidenced by increased avoidance in open field tests (Kamimura et al., 2021). Neurobiologically, GF mice exhibited altered expression of brain-derived neurotrophic factor (BDNF) and ΔFosB in the prefrontal cortex (PFC), a region critical for stress and emotional regulation, thereby affecting the gut-brain axis (Kamimura et al., 2021). Cohabitation with specific pathogen-free mice resulted in the normalization of these behaviours, indicating a pivotal role for the gut microbiota in regulating anxiety and related neurobiological pathways (Kamimura et al., 2021).

#### 4.3.5 Studying AD using the GF mice

Recent studies have highlighted the significant role of gut microbiota in modulating AD pathologies and cognitive disorders through neuroinflammation associated with polyunsaturated fatty acids (PUFAs). A study conducted on GF mice, which were recolonized with fecal samples from both AD patients and healthy donors, revealed a substantial reduction in cerebral amyloid-*β* plaques and neurofibrillary tangles when compared to specific-pathogen-free mice (Chen et al., 2022). This underscores the importance of a complex gut microbiome for the emergence of behavioural abnormalities and AD pathologies. The study identified an enrichment of bacteroides in the gut microbiota composition, which mediates proinflammatory PUFA metabolism, thereby activating microglia and promoting neuroinflammation in the brain. Metabolomic analysis revealed elevated levels of PUFA metabolites and oxidative enzymes, including cyclooxygenases (*COX-1*, *COX-2*) and 5-lipoxygenase, which are linked to inflammation and cognitive impairments (Chen et al., 2022). The activation of the C/EBP*β*/asparagine endopeptidase (AEP) pathway was notably observed, further associating gut dysbiosis with exacerbated AD pathology and cognitive dysfunctions (Chen et al., 2022). To achieve a near-germ-free status, researchers administered a short-term antibiotic to deplete the gut microbiota in APPSWE/PS1ΔE9 transgenic mice (Peng et al., 2009; Tong et al., 2016). This was followed by FMT from aged AD mice donors. The study revealed that this microbiota reconstitution resulted in increased amyloid-beta plaque formation and altered astrocyte activation around the plaques, rather than affecting microglia. The study identified several key biomolecular changes, including the suppression of astrocytic complement component C3 and alterations in astrocyte morphology (Wang et al., 2021). The findings indicate the significance of the microbiota in Neuroimmune and neuroinflammatory, which are summarized in Table 3.

**Table 3:** Neuroimmune and neuroinflammatory disorders studied in GF mice.

Condition		Gut microbiota status	Observed effect	Genes/mechanisms	Reference
Movement disorder	PD	GF mice	↓ Dopamine levels in frontal cortex, striatum, hippocampus; ↓ TH mRNA in cerebellum; ↓ TH protein in striatum; ↓ TH immuno-positive cells and nerve fiber density in striatum	Dopamine, TH	Wang et al. (2023)
ND	ASD	GF mice with ASD microbiota	Behavioural changes, gastrointestinal alterations, ↑ anxiety-like behaviors, altered gene expression	Cntnap2, Shank3, Gabrb3, *Bifidobacterium* (↓), *Lactobacillus* (↑), *Desulfovibrio* (↑)	Berding and Donovan (2019)
Cognitive disorders	AD	GF 3 × Tg mice recolonized with AD microbiota	↓ Cerebral amyloid-β plaques, ↓ neurofibrillary tangles, ↑ Bacteroides, ↑ proinflammatory PUFA metabolism, ↑ neuroinflammation	COX-1, COX-2, 5-LOX, C/EBPβ, AEP, Aβ, C3 complement component	Wang et al. (2021) and Chen et al. (2022)
	CJD	GF mice	No changes in neuropathological features, No significant differences in microglial density or morphology	GFAP, Iba1, SCFAs	Bradford et al. (2017)
Stress response-diseases	CD	GF mice with CD microbiota	↑ Susceptibility to Crohn’s-like symptoms, ↑ immune response, ↑ inflammatory pathways, ↑ immune cell infiltration	CD74, TNF, cytokine receptors	Sheikh et al. (2024)
	Anxiety	GF mice	↑ Anxiety-like behaviors, altered expression of BDNF, ΔFosB	BDNF, ΔFosB	Kamimura et al. (2021)

Parkinson’s disease (PD), autism spectrum disorder (ASD), neurodevelopmental disorder (ND), Alzheimer’s Disease (AD), Creutzfeldt-Jakob disease (CJD), Germ-free (GF), Crohn’s disease (CD), Cluster of Differentiation 74 (CD74), Tumour necrosis factor (TNF), Brain-derived neurotrophic factor (BDNF), Protein fosB (ΔFosB), Tyrosine Hydroxylase (TH), amyloid-beta (Aβ), polyunsaturated fatty acid (PUFA), Complement component 3 (C3), Short-chain fatty acids (SCFAs), contactin-associated protein-like 2 protein (Cntnap2), SH3 and multiple ankyrin repeat domains 3 (Shank3), Gamma-aminobutyric acid type A receptor 3 (GABRb3), asparagine endopeptidase (AEP), cyclooxygenase-1 and-2 (COX-1, and-2), 5-lipoxygenase (5-LOX), CCAAT/enhancer binding protein (C/EBP), Glial Fibrillary Acidic Protein (GFAP), and Ionized calcium-binding adapter molecule (Iba1).

#### 4.3.6 Studying CJD using the GF mice

Creutzfeldt–Jakob disease (CJD) is a neurologic disorder caused by the buildup of misfolded prion proteins (PrPSc) in the brain, resulting in spongiform degeneration, and astrogliosis. Common symptoms of CJD include rapidly worsening dementia, myoclonus, visual impairments, and ataxia (Nakhal et al., 2024). In prion disease patients, there is a reduction in SCFAs due to decreased *Prevotellaceae* (Guo et al., 2022; Salim et al., 2023). Increased levels of *Fusobacteria* have also been observed in CJD patients (Sparks Stein et al., 2012; Bhattacharyya et al., 2016). A study reported that the absence of the commensal gut microbiota in germ-free mice did not affect the susceptibility to prion disease (Bradford et al., 2017). The distribution of neuropathological hallmarks of terminal prion disease, such as spongiform pathology, accumulation of PrPSc, astrogliosis, and microglial activation, were similar in both conventionally housed mice with a typical microbiota composition and germ-free mice (Bradford et al., 2017). During CNS prion disease, microglial activation is anti-inflammatory, aiding in prion clearance. The study found that microbiota absence minimally affected microglial abundance or complexity, indicating their limited role in microglial behavior during prion disease (Bradford et al., 2017). The influence of the microbiota on microglial function may depend on their activation status and phenotype.

## 5 The microbiota influences the brain by producing SCFAs

**Figure 5:**
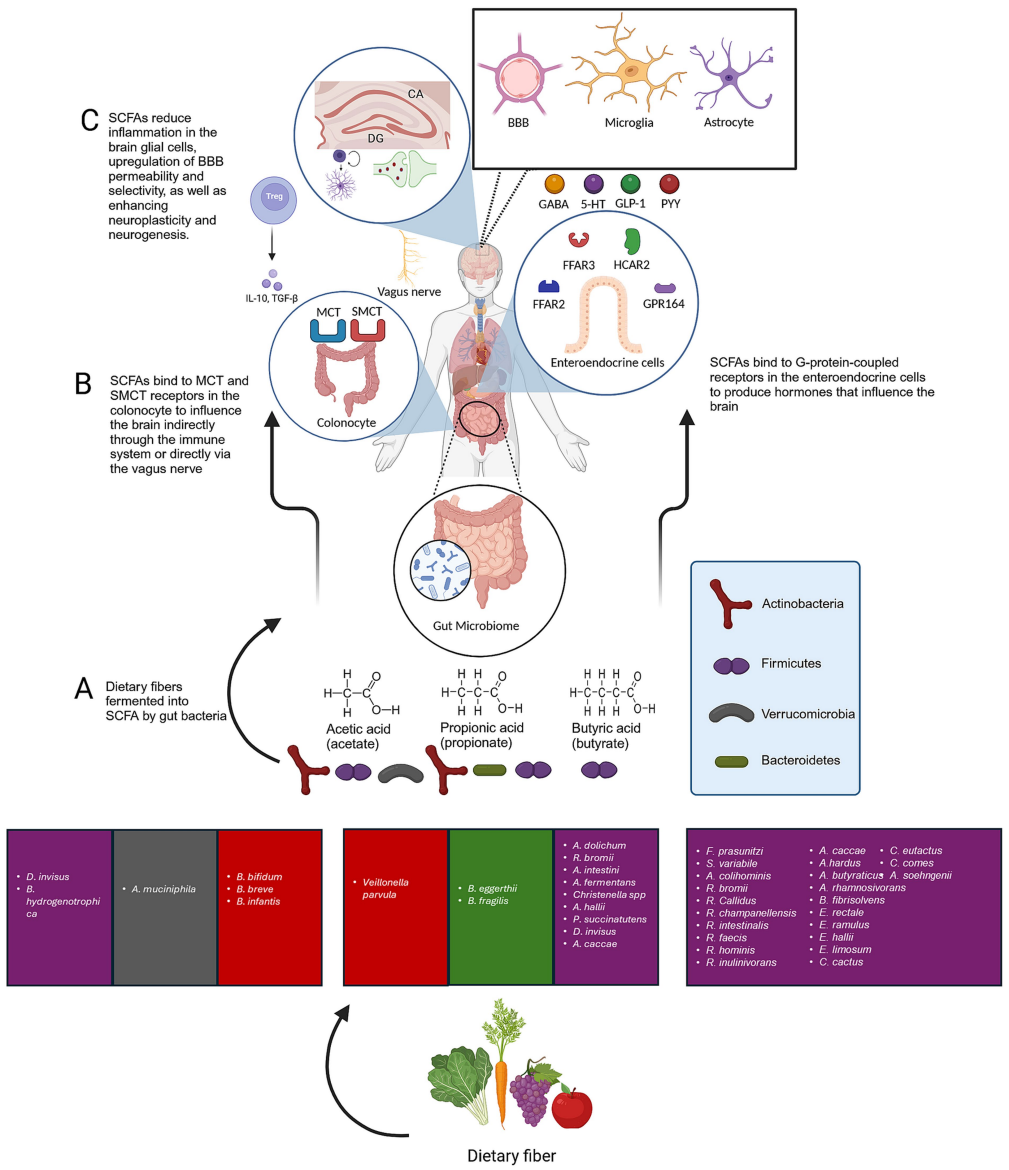
The role of SCFAs produced by gut bacteria in overall brain structure and function. **(A)** The primary metabolites generated in the colon through bacterial fermentation of dietary fibers and resistant starch are acetate, propionate, and butyrate**. (B)** SCFAs are absorbed by colon cells, primarily via MCTs and SMCTs. This process allows for the direct influence of SCFAs on the brain through the vagus nerve. SCFAs that are not utilized by colon cells are transported into the. Moreover, SCFAs interact with GPCRs. Well-researched SCFA receptors include FFAR2 and FFAR3, as well as HCAR2 and GPR164. These receptors are expressed in a variety of tissues, including the GI mucosa, the CNS, and the immune system. SCFAs can influence the brain indirectly by binding to their receptors on enteroendocrine cells, thereby stimulating the production of GLP-1 and PYY. However, when binding to *β*-pancreatic cells, it causes an increase in insulin output. Furthermore, SCFAs can reduce inflammatory signalling in the CNS by influencing the immune system at the cellular level. This is achieved by regulating overall systemic functions and inhibiting the activity of HDAC, which leads to the acetylation of lysine residues on nucleosomal histones and the release of IL-4, IL-6, IL-10, and IL-11, furthermore, it has been well-documented that butyrate can induce regulatory T cell (Treg) differentiation and regulate inflammation. **(C)** SCFAs improve BBB selectivity, microglia maturation while reducing microglial activation and the release of pro-inflammatory cytokines by lowering the production of IL-1β, IL-6, and TNF-α, as well as the phosphorylation of p38 MAPK, JNK, and NF-κB. Acetate can regulate pro-inflammatory pathways, inflammatory cytokines signalling, activation, and proliferation of primary astrocytes. SCFAs enhance neuroplasticity and neurogenesis in the brain. Created with BioRender.com.

The precise methods of communication between the gut microbiota and the CNS remain elusive. Nevertheless, several potential mechanisms have been proposed through which the bacteria in the gut may influence brain function (Borre et al., 2014). One pathway stands out significantly, namely the production of SCFAs with neuroactive characteristics (Tan et al., 2014; Fung et al., 2017; Dalile et al., 2019). The primary metabolites generated in the colon through bacterial fermentation of dietary fibers and resistant starch are acetate, propionate, and butyrate, along with additional minor metabolites such as lactate, valerate, and formate (Pascale et al., 2018). In addition to their established roles in energy provision and T regulatory cell regulation, there is mounting evidence suggesting that SCFAs play a pivotal role in influencing the physiological functions of the brain (Stilling et al., 2016; Dalile et al., 2019) (Figure 5). A number of studies in animals and humans have proposed the concept of manipulating the microbiota and administering SCFAs as potential key candidate treatments for neurological disorders such as depression, AD, PD, and ASD (Stilling et al., 2016; Zhang et al., 2017; Deng et al., 2019; Sharon et al., 2019).

### 5.1 SCFAs absorption and mechanism of action

Following the production of SCFAs by microbial bacteria (Figure 5A), they are absorbed by colon cells, primarily via H+ − dependent (MCTs) or sodium-dependent monocarboxylate transporters. This process allows for the direct influence of SCFAs on the brain through the vagus nerve (Vijay and Morris, 2014). SCFAs that are not utilized by colon cells are transported into the portal vein and serve as an energy source for liver cells, with the exception of acetate, which is not metabolized in hepatocytes (Schönfeld and Wojtczak, 2016). Moreover, SCFAs interact with G protein-coupled receptors. Well-researched SCFA receptors include free fatty acid receptor (FFAR2) and FFAR3, as well as GPR109a/HCAR2 (hydrocarboxylic acid receptor) and GPR164 (Figure 5B). These receptors are expressed in a variety of tissues, including the GI mucosa, the CNS, and the immune system (Bolognini et al., 2016; Mohajeri et al., 2018). The outcome of stimulating such receptors varies considerably depending on the location of expression. For instance, SCFAs can influence the brain indirectly by binding to their receptors on enteroendocrine cells, thereby stimulating the production of glucagon-like peptide 1 and peptide YY (Cherbut et al., 1998). However, when binding to β-pancreatic cells, it causes an increase in insulin output (Puddu et al., 2014). Furthermore, SCFAs can reduce inflammatory signalling in the CNS by influencing the immune system at the cellular level. This is achieved by regulating overall systemic functions and inhibiting the activity of histone deacetylase, which leads to the acetylation of lysine residues on nucleosomal histones and the release of interleukins such as IL-4, IL-6, IL-10, and IL-11, furthermore, it has been well-documented that butyrate has the ability to induce regulatory T cell differentiation and regulate inflammation (Arpaia et al., 2013; Smith et al., 2013; Haghikia et al., 2015; Fung et al., 2017).

### 5.2 Role of SCFAs role in gut-brain axis

SCFAs have been linked with an essential role in the communication between the microbiota and the brain. This is evidenced by the fact that all three main metabolites (acetate, propionate, and butyrate) can be identified in human cerebrospinal fluid (Vijay and Morris, 2014). Acetate concentrations typically range from 0 to 171 μM, propionate from 0 to 6 μM, and butyrate from 0 to 2.8 μM, with a molar ratio of approximately 60:20:20 (Human Metabolome Database, 2023). Animal studies in rats and mice have demonstrated that the levels of butyrate in the brain increase significantly when supplemented with live bacteria such as *Clostridium butyricum*. Moreover, the high levels of monocarboxylate transporters in endothelial cells facilitate the crossing of SCFAs across the BBB, as evidenced by previous studies in which rats absorbed 14C-SCFAs injected into the carotid artery (Oldendorf, 1973; Kekuda et al., 2013; Liu et al., 2015; Sun et al., 2016). As demonstrated in (Figure 5), the role of bacteria-produced SCFAs is further elucidated.

### 5.3 SCFAs and BBB modifications

SCFAs not only penetrate the BBB, but they also help maintain its integrity, which is crucial for regulating the transport of substances and nutrients from the bloodstream to the brain. This process is essential for brain growth and maintaining the balance of the CNS (Silva et al., 2020). This claim was supported by GF mice, which exhibited decreased levels of tight junction proteins, including claudin and occludin, resulting in elevated permeability of the BBB throughout prenatal development to adolescence. Nevertheless, the BBB quality was retrieved after recolonization with SCFAs-producing bacteria. Given that most neurological diseases are linked with BBB disruption and decreased selectivity, butyrate has been shown to attenuate the behavioral assessment results of neurological disorders by improving BBB selectivity (Downs et al., 2014).

### 5.4 SCFAs influencing microglia

Microglial cells have been demonstrated to play a pivotal role in the removal of superfluous or unnecessary synaptic connections, which is vital for the development of interconnections in the nervous system (Liu et al., 2015; Wilton et al., 2019). Consequently, it appears that gut microbiota bacteria exert a pivotal influence on the formation and functionality of the innate immune system within the CNS, through their impact on the maturation and functionality of microglial cells (Erny et al., 2015). This was corroborated by the observation that GF animals exhibited abnormalities in microglia, including changes in cell morphology and an immature phenotype, which resulted in compromised innate immune reactions. Supplementation of acetate, propionate, and butyrate to GF mice resulted in reversal of microglial characteristics and responsiveness, accompanied by improvement in maturation (Stilling et al., 2016; D’Alessandro et al., 2022). The hypothesis is that the pathway of microglial maturation through SCFAs produced by gut bacteria is through the activation of FFAR2, which results in the inhibition of Histone deacetylases (HDACs). This is based on the observation that mice lacking FFAR2 receptors in the GI mucosa showed similar microglial malfunction and phenotype to those in GF mice (Gautier et al., 2012). Subsequently, antibiotics have been shown to induce depletion of the gut microbiota in experimental animals, resulting in increased neurological inflammation and changes in microglial morphology towards a pro-inflammatory state (Minter et al., 2017; Jang et al., 2018). However, it is noteworthy that sodium butyrate has been demonstrated to possess the capacity to reduce microglial activation and the release of pro-inflammatory cytokines in a range of neurological disorders (Wang et al., 2015; Patnala et al., 2017; Yamawaki et al., 2018). Similarly, similar results were observed in other SCFAs, such as acetate. Microglial culture treatment with acetate has been shown to decrease the expression of inflammatory signals by lowering the production of IL-1β, IL-6, and TNF-*α*, as well as the phosphorylation of p38 MAPK, JNK, and NF-κB (Soliman et al., 2012).

### 5.5 SCFAs influencing neuronal function

Microbial metabolites contribute to neuronal function, as evidenced by the reported regulation of neurotransmitter and neurotrophic factor levels by SCFAs through the HPA axis (Silva et al., 2020). For example, acetate has been shown to alter the levels of neurotransmitters, including glutamate, glutamine, and GABA, in the hypothalamus, and to enhance the expression of anorexigenic neuropeptides (Frost et al., 2014). It has been demonstrated that propionate and butyrate modulate intracellular potassium levels, thereby suggesting that SCFAs are involved in cell signalling systems (Oleskin and Shenderov, 2016). SCFAs exert their influence on the brain neurons through a variety of mechanisms. For instance, they regulate the levels of tryptophan 5-hydroxylase 1, an enzyme involved in serotonin production, and tyrosine hydroxylase, which is essential for the synthesis of dopamine, noradrenaline, and adrenaline. This enables them to influence cerebral neurochemistry (Clarke et al., 2014; Nankova et al., 2014; Reigstad et al., 2015; Yano et al., 2015). Additionally, SCFAs have been demonstrated to modulate neurotrophic factors, including nerve growth factor, glial cell line-derived neurotrophic factor, and BDNF. These factors are involved in the development, viability, proliferation, and differentiation of neurons and synapses in the brain (Intlekofer et al., 2013; Savignac et al., 2013; Barichello et al., 2015; Varela et al., 2015). In a corresponding manner, all three SCFAs were observed to enhance the survival and growth of human brain progenitor cells and to promote mitosis. These findings provide insights into the potential role of SCFAs in regulating early nervous system development and hippocampal neurogenesis (Levenson et al., 2004; Yang et al., 2020). The significance of SCFAs in shaping the CNS development is well established. Emerging evidence indicates that SCFAs can attenuate long-term memory, cognition, and anxiety in various neurodevelopmental and neurodegenerative diseases (Wang et al., 2012; Dinan and Cryan, 2017; Kelly et al., 2017; Ho et al., 2018; Skonieczna-Żydecka et al., 2018).

### 5.6 SCFAs influencing astrocytes

Astrocytes are the predominant cells in the CNS of humans, considered as a subtype of glial cells (Parpura et al., 2012). They provide reduction against glutamate toxicity, glucose induced stress, and redox stress (Bolaños, 2016). It is well documented that astrocytes lose their homeostatic functions and gain toxic functions in neurodegenerative diseases (Valori et al., 2019). Nonetheless acetate has shown the ability to regulate pro-inflammatory pathways, inflammatory cytokines signalling, activation, and proliferation of primary astrocytes, which elucidates the attenuating effects of experimental models induced neurological disorders when supplemented with acetate (Soliman et al., 2013). In contrast, there is a lack of evidence to the role of propionate and butyrate in modulating astrocyte’s function.

### 5.7 SCFAS role in brain disorders

A number of studies have demonstrated that the composition of the gut microbiome and metabolome is altered in individuals with various brain disorders (Hill et al., 2014; Li et al., 2016; Unger et al., 2016; He et al., 2018; Zhai et al., 2019). Consequently, the targeting of gut bacteria metabolites for the prevention or mitigation of CNS pathologies progression represents a promising avenue for future research (Wang et al., 2012; Dinan and Cryan, 2017; Kelly et al., 2017; He et al., 2018; Skonieczna-Żydecka et al., 2018). In accordance with this hypothesis, clinical studies have indicated that the diagnosis and severity of ASD are correlated with the composition of SCFAs-producing bacteria in human stool. Specifically, butyrate-producing bacteria levels were found to be considerably low, while propionate-producing bacteria were increased (Finegold et al., 2010; Finegold, 2011). Consequently, one of the validated experimental animal models of autism is the induction of autism by propionate. The administration of elevated amounts of propionate to different animal models via the subcutaneous, intragastric, intraperitoneal, or intracerebroventricular routes has been shown to initiate microglia abnormal stimulation, neurotoxic cytokine output, genetic expression modifications, atypical hippocampal histology, and ASD-like behaviours, including repetitive movement, increased anxiety, and impaired social cooperation (Choi et al., 2018). Conversely, butyrate has been shown to mitigate the behavioural outcomes of Black and Tan BRachyury (BTBR) mice with ASD-like symptoms (Kratsman et al., 2016). The aforementioned improvements may result from HDAC inhibition, which leads to epigenetic changes and regulates the transcription of inhibitory neurotransmitters in the brain’s PFC (Kratsman et al., 2016). Additionally, enhancing BBB integrity may contribute to these improvements (Delgado, 2000). Moreover, depression is a highly prevalent mood disorder that is characterized by social impairments and a high risk of mortality. It is typically associated with a deficiency of monoamines, disturbances in neurogenesis, and elevated levels of inflammatory biomarkers in the brain (Miller and Raison, 2016; Szczesniak et al., 2016). In accordance with these findings, clinical reports have illustrated that stool SCFAs levels were significantly lower in patients diagnosed with depression in comparison with healthy controls (Valvassori et al., 2016; Skonieczna-Żydecka et al., 2018). Other animal models of depression exhibited a similar fecal composition and low SCFAs levels (Deng et al., 2019). SCFAs have also been associated with improving behavioural assessment of the mouse model of depression. This is evidenced by the induction of antidepressant-like benefits, higher energy levels, decreased anhedonia, improved sociability, and cognitive functions (Wei et al., 2015; Burokas et al., 2017). SCFAs have demonstrated efficacy in the treatment of mood disorders, with evidence suggesting their ability to reverse social impairment and reduce stress-induced corticosterone release (Van De Wouw et al., 2018). Additionally, they have been shown to decrease manic attacks and depressive-like characteristics in rats (Resende et al., 2013). Butyrate demonstrated anti-manic properties in a rat model of bipolar disorder induced by ouabain (Valvassori et al., 2016). Conversely, a clinical study of schizophrenic patients has documented elevated levels of SCFA-producing bacteria in their microbiome composition, despite direct evaluation of metabolite levels or the presence of metabolite imbalances (He et al., 2018).

#### 5.7.1 SCFAs and AD

AD is the most prevalent form of dementia. It is characterized by a progressive decline in cognitive function (Prince et al., 2016). Several studies have demonstrated the benefits of maintaining a balanced microbiome in slowing the progression of AD. There is also a growing body of evidence indicating a link between dysbiosis and the increasing severity of the disease (Cryan and Dinan, 2012; Hill et al., 2014; Hoffman et al., 2019). In this context, an animal microbiome study reported a decline in SCFAs levels in a mouse model of AD (Zhang et al., 2017). SCFAs act by interrupting the main synapse dysfunction and cognitive impairment toxin known as amyloid-*β* peptides, which disrupt their progression into neurotoxic molecules (Ho et al., 2019). This notion is consistent with previous research demonstrating the positive impacts of butyrate and probiotic therapy on cognitive function and memory in D-galactose aging mice and rats models, which are associated with the development and advancement of AD (Garcez et al., 2018; Ho et al., 2019). Finally, butyrate supplementation improved memory function and elevated the expression of genes associated with cognitive learning in the APP/PS1 mice model of AD by inhibiting HDAC (Govindarajan et al., 2011).

#### 5.7.2 SCAFAs and PD

The role of SCFAs in PD is a topic of contention. PD is a complex, multifactorial disorder that presents with tremors, stiffened muscles, bradykinesia, and distorted movement. These symptoms result from *α*-synuclein (αSyn) aggregation, which downregulates dopaminergic neurons. The sequencing results of the stool microbiota of PD patients indicated a reduced population of propionate-producing bacteria species, as well as increased amounts of Enterobacteriaceae, a family known to deplete SCFAs production when compared with healthy controls (Unger et al., 2016). Nevertheless, the presence of gut microbiota is of substantial importance for αSyn abnormal expression, as evidenced by the findings of gut dysbiosis models, which demonstrated an improvement in disease progression. The intricate composition of the microbiome is either dominated by bacterial strains that ferment fibre and provide SCFAs, which ameliorate PD pathophysiology, or by bacterial strains that degrade fibre, thereby exacerbating PD progression and development due to excessive synthesis of endotoxins and neurotoxins (Li et al., 2017). In accordance with these reports, the administration of butyrate to animal models of Parkinson’s disease has been observed to enhance motor function and alleviate dopamine depletion (Laurent et al., 2013; Sharma et al., 2015; Liu et al., 2017; Paiva et al., 2017).

### 5.8 SCFAs producing bacteria

The pivotal role of these biochemical messengers produced by intestinal bacteria in CNS development and homeostasis has been established (Silva et al., 2020). Whole-genome sequencing and metabolomics have identified some of the bacteria that produce SCFAs. The production of SCFAs can occur directly from bacterial fermentation or by cross-feeding from other SCFAs, such as acetate, which assists the growth of propionate (Fusco et al., 2023). The primary species responsible for butyrate production are *Lachnospiraceae* and *Ruminococcaceae*, as well as other families such as *Lactobacillaceae*, *Unclassified Clostridiales* 5, and *Clostridiaceae*, which are predominantly classified to the *Firmicutes* phylum (Singh et al., 2023). Nevertheless, a few species belonging to *Actinobacteria*, *Fusobacteria*, and *Proteobacteria* are also capable of producing butyrate (Vital et al., 2014; Anand et al., 2016). The *Ruminococcaceae* family contains *Faecalibacterium prausnitzii*, which constitutes between 5 and 17% of the microbiota found in the feces of healthy adults. This bacterium is one of the most prevalent in the production of butyrate (Miquel et al., 2013; Martín et al., 2018). Other species, including *Anaerobutyricum hallii*, *Eubacterium rectale*, *Lactobacillus casei*, *Coprococcus eutactus*, *Coprococcus comes*, *Butyricicoccus pullicaecorum*, *Roseburia intestinalis*, and *Clostridium butyricum*, were also found to produce butyrate in healthy individuals (Fusco et al., 2023). An example of cross-feeding is observed when *Akkermansia muciniphila* stimulates the release of oligosaccharides and acetate to feed *Anaerobutyricum hallii*, thereby generating propionate and butyrate (Belzer et al., 2017; Geerlings et al., 2018; Paone and Cani, 2020). Additionally, increased butyrate production through cross-feeding is observed among *Faecalibacterium prausnitzii* and *Bifidobacterium adolescentis* (Fusco et al., 2023). Moreover, genomic studies in animal models and healthy humans have indicated that an increase in Bifidobacteria is associated with elevated SCFAs levels (Wang et al., 2014; Hor et al., 2019; Pérez-Burillo et al., 2020). In particular, *B. bifidum*, *B. infantis*, and *B. breve* are the primary acetate producers within the *Bifidobacteriaceae* family (Matsuki et al., 2016; Tsukuda et al., 2021), in addition to *Dialister invisus* and *Akkermansia muciniphila*, which are capable of producing both acetate and propionate (Nagao-Kitamoto and Kamada, 2017; Rodrigues et al., 2022). Other documented propionate-producing bacteria include *Phascolarctobacterium succinatutens*, *Anaerobutyricum hallii*, *Anaerostipes caccae*, *Coprococcus eutactus*, *Coprococcus comes*, and *Prevotella* spp. (Watanabe et al., 2012; Reichardt et al., 2014; Salonen et al., 2014; De Vadder et al., 2016).

## 6 Bacteria strains influencing brain function

### 6.1 Actinobacter phylum

The abundance of *Acinetobacter calcoaceticus* in patients with MS was found to be higher than in healthy participants (Cekanaviciute et al., 2017). The mechanism of action of *Acinetobacter calcoaceticus* has been demonstrated to promote MS by inducing pro-inflammatory cytokines and Th1, while blocking T regulatory cells in humans and mice (Chatterjee and Banerjee, 2020). Conversely, not all species from the Actinobacteria phylum are linked with deteriorating effects. For instance, *Bifidobacterium bifidum* (Martínez Pizarro, 2022), *Bifidobacterium longum* (Pinto-Sanchez et al., 2017), and *Bifidobacterium breve* (Głaz et al., 2023), it was demonstrated that this bacterium decreases depression scores and mitigates major depression disorder by modulating the gut flora and tryptophan metabolism. This could be a potential target for depression treatment or prevention (Li et al., 2023). Furthermore, a reduction in the population of gut microbiota belonging to the Bifidobacterium spp. was observed in AD patients when compared to healthy controls (Chandra et al., 2023). A taxonomic analysis of the Actinobacteria phylum was conducted in schizophrenic patients, revealing an increase in the abundance of *Bifidobacterium dentium*, *Collinsella aerofaciens*, and *Bifidobacterium adolescentis*, while *Bifidobacterium bifidum*, *Bifidobacterium longum*, and *Bifidobacterium breve* exhibited a decrease (Kraeuter et al., 2020). In accordance with previous studies, two-year-old mice exhibited a reduction in beneficial bifidobacteria (*Bifidobacterium bifidum*, *Bifidobacterium breve*, *Bifidobacterium infantis*, *Bifidobacterium longum*) in comparison with their fecal composition at a younger age. Furthermore, colonization with a mixture of infant-type bifidobacterium strains has been shown to reverse cognitive deficits in GF mice (Luk et al., 2018). Their abundance is negatively correlated with pro-inflammatory cytokines in humans (Hébuterne, 2003). In a mouse model of AD, supplementation with SLAB51, which includes *Bifidobacterium longum*, *Bifidobacterium breve*, and *Bifidobacterium infantis*, restored neuroplasticity by acting on neuronal proteolytic pathways, such as autophagy and the ubiquitin-proteasome system. Additionally, it reduced oxidative stress biomarkers in the brain (Bonfili et al., 2017). In humans, supplementation with *Bifidobacterium animalis* has been linked with reduced psychiatric symptoms and anxiety occurrence (Severance et al., 2017). Furthermore, an fMRI study demonstrated that *Bifidobacterium longum* administration resulted in a reduction in the reactivity to negative emotional stimuli in various brain regions, including the amygdala and fronto-limbic areas, when compared to the placebo group (Pinto-Sanchez et al., 2017). In a similar vein, the administration of *Bifidobacterium longum* has been observed to upregulate hypothalamic BDNF, which in turn promotes neural plasticity and neurogenesis in a brain stress-induced model of mice (Ait-Belgnaoui et al., 2014). Consequently, the brain alterations associated with *Bifidobacterium longum* have been demonstrated to result in behavioural and cognitive ameliorations in clinical and experimental studies. These include stress attenuation, anxiety reduction, and improved memory performance in healthy humans and AD patients (Allen et al., 2016). Additionally, elevated fear learning and short-term memory have been observed in anxious adult mice (Savignac et al., 2015) It was demonstrated that *Bifidobacterium longum* could improve the behavioural symptoms of autistic children (Grimaldi et al., 2018). Furthermore, it has been shown to decrease depression scores in individuals with mild to moderate MDD (Kazemi et al., 2019) and IBS patients (Pinto-Sanchez et al., 2017). Finally, *Bifidobacterium animalis* has been shown to restore cognition and memory in an AD rat model (Athari Nik Azm et al., 2018). The anxiolytic impact observed in this study could be attributed to the ability of the compound to regulate the HPA axis response, as evidenced by previous studies in mice, rats, and humans (Messaoudi et al., 2011; Ait-Belgnaoui et al., 2014). This effect involves the regulation of CNS and ENS hormone secretion homeostasis. Inulin and omega-3 have both been demonstrated to enhance brain function, and there is also evidence that they can increase the population of bifidobacteria in the microbiome (De Preter et al., 2008; Robertson et al., 2017). In a study conducted by Luk et al. (2018), it was observed that colonization of bifidobacteria in adult mice resulted in enhanced anxiety, cognition, locomotion, and memory in infants (Luk et al., 2018). This evidence suggests that the microbiome plays a pivotal role in the process of aging. The Human Microbiome Project has proposed that the microbiome found in faeces has the ability to generate glutamate decarboxylase, an enzyme that transforms glutamic acid into gamma-aminobutyric acid (GABA) (Pokusaeva et al., 2017). Similarly, a study reported that *Bifidobacterium adolescentis* from the human gut (Barrett et al., 2012) was able to convert monosodium glutamate into GABA. This process was also observed in rats’ hippocampus after gut dysbiosis caused by antibiotics (Liang et al., 2017). *Bifidobacterium adolescentis* has been linked with the reduction of depression, anxiety, and improvement of sleep quality in humans (Lee et al., 2021). These findings show varying effects of Actinobacteria on the brain, with species-specific variations in benefits and detriments.

### 6.2 Firmicutes phylum

*Faecalibacterium prausnitzii* has been demonstrated to exhibit anti-inflammatory, anxiolytic, and antidepressant therapeutic benefits in rats. These benefits are achieved by increasing butyrate production and IL-10 while reducing corticosterone and IL-6 (Hao et al., 2019). Inulin administration has been shown to enhance *Faecalibacterium prausnitzii* growth (Ramirez-Farias et al., 2008). Furthermore, a clinical study involving over 1,000 participants demonstrated that individuals with depression and low quality of life exhibited a lower abundance of species belonging to the Faecalibacterium and Coprococcus genera when compared with healthy controls (Valles-Colomer et al., 2019). An analysis of fecal microbiota revealed reduced levels of Firmicutes, particularly Faecalibacterium, in individuals with bipolar disorder (Evans et al., 2017). In a separate study, fecal samples from Chinese patients with transient ischemic attacks exhibited significantly reduced levels of Faecalibacterium in their fecal samples (Yin et al., 2015). The levels of *Faecalibacterium prausnitzii* are negatively correlated with aging, as this bacterium has been associated with reducing frailty in geriatrics (Bartosch et al., 2004; Van Tongeren et al., 2005). The abundance of *Faecalibacterium prausnitzii* is reduced in individuals with mild cognitive impairment, and specific strains of *Faecalibacterium prausnitzii* obtained from healthy volunteers have been demonstrated to enhance cognitive function in mice with cognitive impairment. A comprehensive metagenome and whole-genome analysis has identified these strains as potential candidates for interventions targeting the gut microbiome in AD. It can be seen that there is a difference in the levels of *Clostridium coccoides* present in the stool samples of patients with PD and those of healthy controls (Ueda et al., 2021). This is in accordance with the findings of Hasegawa et al., 2015, where the abundance of *Clostridium coccoides* was found to be significantly lower in the stool samples of patients with PD than in those of healthy controls (Hasegawa et al., 2015). The colonization of *Clostridium coccoides* into GF mice resulted in a decrease in anxiety levels, while having no significant impact on locomotor activity (Nishino et al., 2013). Such behavioural modifications may be attributed to the enzymatic activity of beta glucuronidase, which enables *Clostridium coccoides* and *Clostridium leptum* to alter dopamine into its active form (Asano et al., 2012).

Some species from the Firmicutes phylum exhibit both commensal and pathogenic characteristics. *Clostridioides difficile*, for instance, is known to induce mental confusion and brain fog as a result of dopamine metabolism dysregulation (Vinithakumari et al., 2021). This bacterium is prevalent in autistic children, although its presence has been associated with the use of intense antibiotic regimens that destroy beneficial bacteria and allow *Clostridioides difficile* to flourish (Kakabadze et al., 2022). The expression of IL-10 in mice was found to be associated with a higher presence of Clostridium spp., suggesting that there may be a collaborative relationship between the gut bacteria, the adaptive immune system, and the CNS (Smith et al., 2014). Conversely, certain species belonging to the Blautia genus, including *Blautia coccoides* and *Blautia hydrogenotrophica*, which are responsible for the production of propionate and acetate, respectively, have been linked to 5-HT production. In a mouse model of autism, these bacteria have been observed to display pro-5-HT activity (Golubeva et al., 2017). In addition to the modulation of 5-HT production in humans and rodents by *Clostridium perfringens* (Beaver and Wostmann, 1962). *Clostridium butyricum* has been demonstrated to confer anti-depressive benefits in a chronic stress mouse model (Sun et al., 2018). A taxonomic analysis revealed a decline in lactobacillus species with age, suggesting a potential correlation with age-related neurodegenerative diseases (Hébuterne, 2003). *Lactobacillus reuteri* has been shown to improve behavioural examination results in a mouse model of ASD, including sociability, social novelty, and reciprocal social interaction (Sgritta et al., 2019).

*Lactobacillus acidophilus*, *Lactobacillus reuteri*, *Lactobacillus casei*, *Lactobacillus delbrueckii subsp. bulgaricus*, and *Lactobacillus brevis* have been demonstrated to produce GABA and glucagon-like peptide-1. However, GF mice exhibited lower levels of these metabolites when compared with control groups (Li et al., 2008; Zareian et al., 2012; Simon et al., 2015; Panwar et al., 2016). In accordance with this hypothesis, Lactobacillus strains have been extensively employed for this purpose as probiotic treatments. A variety of strains from the Lactobacillus family have been demonstrated to improve object recognition memory impairments caused by continuous restraint stress (Liang et al., 2015), as well as to avoid these impairments in GF mice (Gareau et al., 2011), a mouse model of colitis (Emge et al., 2016), diabetic mice (Davari et al., 2013), and immunodeficient mice (Smith et al., 2014). It is noteworthy that the administration of lactobacillus strains has been shown to improve cognitive test results in healthy adults (Chung et al., 2014) and AD patients (Akbari et al., 2016), in comparison with a placebo group. Conversely, a high abundance of lactobacillus has been observed in PD patients (Hasegawa et al., 2015). The strains were successful in correcting spatial memory impairments caused by numerous factors in mice. Furthermore, the depletion of species from the Lachnospiraceae and Lactobacillus families has been observed in human MDD (Naseribafrouei et al., 2014; Aizawa et al., 2016). *Lactobacillus farciminis* and *Lactobacillus salivarius* have been observed to inhibit the overstimulation of the HPA axis during acute stress in rats. This effect may be attributed to their ability to prevent excessive permeability of the gut, which has been linked to benefits in the reduction of aggression, anxiety, and depression-related disorders (Ait-Belgnaoui et al., 2012; Bagga et al., 2018). *Lactobacillus acidophilus*, *Lactobacillus plantarum*, *Lactobacillus casei*, and *Lactobacillus delbrueckii subspecies Bulgaricus* have been demonstrated to improve age-related impairments in LTP, upregulate hippocampal BDNF, and downregulate pro-inflammatory cytokines, thereby improving neuroplasticity and neurogenesis in aged rats (Distrutti et al., 2014). The administration of *Lacticaseibacillus rhamnosus* during pregnancy has been observed to markedly reduce the incidence of postnatal depression and anxiety in humans (Slykerman et al., 2017). This effect has also been demonstrated in the context of obesity (Sanchez et al., 2017). The administration of a probiotic formulation comprising *Lacticaseibacillus rhamnosus* and *Lactobacillus helveticus* has been demonstrated to mitigate the impact of early-life stress on fear behavior and its protective effects on brain networks during development. This explains their benefits in neurodevelopmental diseases such as ASD (Callaghan et al., 2016; Cowan et al., 2016). Experimental and clinical studies have demonstrated that *Lacticaseibacillus rhamnosus* functions via a G protein-coupled receptor-mediated pathway (Mao et al., 2013). This results in increased vagal and spinal cord nerve bundle firing patterns (Perez-Burgos et al., 2013), which have been observed to have beneficial effects, including the alleviation of anxiety in an impaired adaptive immune system mouse model It was demonstrated that *Lacticaseibacillus rhamnosus* reversed the reduction in GABA levels induced by antibiotics in the hippocampus of juvenile rats (Smith et al., 2014; Liang et al., 2017). Furthermore, in 2011, it was shown that *Lacticaseibacillus rhamnosus* prevented memory dysfunction induced by *Citrobacter rodentium* by regulating the c-fos gene (Gareau et al., 2011). Additionally, it was observed that *Lacticaseibacillus rhamnosus* normalized fear behavior in maternally deprived pups (Cowan et al., 2016). Finally, in 2017, it was demonstrated that *Lacticaseibacillus rhamnosus* improved psychiatric symptoms in humans and males, particularly (Severance et al., 2017). The supplementation *of Lactobacillus reuteri*, initially known as *Lactobacillus fermentum*, has been demonstrated to upregulate NMDAR expression in the CA1 and DG of the rat hippocampus, thereby enhancing memory (Wang et al., 2015). The abundance of the *Roseburia* genus is indicative of a healthy microbiota, given its anti-inflammatory and immune homeostasis properties. It has been demonstrated that characteristics such as abundance are associated with the consumption of healthy unsaturated fatty acids (Devillard et al., 2007). In particular, the *Eubacterium rectale* bacteria from the *Roseburia* genus have been observed to increase with exercise in rats, and they are considered to be a component of a healthy microbiome (Queipo-Ortuño et al., 2013). Furthermore, a reduction in species belonging to the *Vellionella* genus was observed in a genetic model of autism in mice (Tabouy et al., 2018). Some species from the Firmicutes phylum have been identified as harmful bacteria, including *Megasphaera* spp., which has been observed to be present in patients with transient ischemic attacks (Yin et al., 2015). A multitude of studies have demonstrated the effect of species from the Lachnospiraceae family on the brain of humans and experimental animals. Lachnospiraceae levels were found to be significantly lower in human depression (Naseribafrouei et al., 2014) and bipolar patients (Flowers et al., 2017), but increased in sleep fragmentation of mice (Poroyko et al., 2016). Nevertheless, atypical antipsychotics have been shown to significantly increase Lachnospiraceae levels in bipolar cohorts’ microbiomes (Flowers et al., 2017). Such influence may be attributed to SCFAs production (Duncan et al., 2002) and the regulation of brain reactivity to fear in the PFC of humans (Callaghan et al., 2020).

### 6.3 Bacteroidetes phylum

It is well established that protein-rich diets and the Mediterranean diet elevate levels of Bacteroides and Parabacteroides in humans and other experimental models (Wu and Hui, 2011; Marlow et al., 2013; Olson et al., 2018). It has been demonstrated that *Bacteroides fragilis* can influence brain functions through its capsular exopolysaccharide at the cell surface, which affects the ENS (Mao et al., 2013). The Mullen Scales of Early Learning indicate that one-year-old toddlers with higher levels of GM Bacteroides were less likely to be born by C-section and exhibited significantly better cognitive abilities (Carlson et al., 2018). Clinical and experimental studies have indicated that Bacteroides may play a role, particularly *Bacteroides fragilis*, in ameliorating brain reactivity to fear in the PFC of children (Callaghan et al., 2020), reducing anxiety and stress responses in mice (Hsiao et al., 2013), without improving sociability. A taxonomic analysis of neurodegenerative diseases such as MS, AD, and PD revealed significantly reduced levels of *Parabacteroides distasonis* (Cekanaviciute et al., 2017) and *Bacteroides fragilis* (Hasegawa et al., 2015) when compared with healthy controls. This suggests that these bacteria may have anti-inflammatory benefits in various brain regions. Despite the benefits of *Parabacteroides distasonis* and *Bacteroides fragilis*, a cohort study of 37 depressed patients revealed that the overall *Bacteroidales* were relatively higher when compared with healthy controls (Naseribafrouei et al., 2014). VPA-exposed experimental models are known to develop ASD-like symptoms (Nakhal et al., 2023), accompanied by disruption of the GI microbiota, which is characterized by alterations in the *Firmicutes*: *Bacteroidetes* ratio (De Vadder et al., 2014). The microbiome of patients with PD exhibited a striking reduction in species belonging to the *Prevotellaceae* family (Keshavarzian et al., 2015). Furthermore, a reduction in species belonging to the *Prevotella* genus was observed in a genetic mouse model of autism (Tabouy et al., 2018). *Porphyromonas* spp. have been linked to cognitive decline in humans (Bajaj et al., 2012; Collins et al., 2012), as well as aging and increased anxiety in mice (Scott et al., 2017).

### 6.4 Proteobacteria phylum

A significant prevalence of sulphate-reducing bacteria species from the *Desulfovibrio* genus, including *Desulfovibrio desulfuricans* and *Desulfovibrio desulfuricans 2*, has been observed in children with ASD compared to healthy controls (Karnachuk et al., 2021). In another study, species from the Enterobacteriaceae family were found to be increased in PD cohorts and their levels were positively correlated with postural instability and dynamic (Keshavarzian et al., 2015). In accordance with these findings, *Enterobacter* and *Desulfovibrio* spp. were prevalent in Chinese patients with transient ischemic attack (Yin et al., 2015). Additionally*Escherichia coli* are known to produce biogenic amines, which are frequently observed in neurological disorders (Pugin et al., 2017).

### 6.5 Tenericutes phylum

The prevention of sudden epileptic seizures was found to be modulated by microbiota changes resulting from the ketogenic diet, with an increase in *Erysipelotrichaceae* levels (Olson et al., 2018). It is noteworthy that newly diagnosed patients with PD who had not yet received levodopa displayed low levels of *Erysipelotrichaceae* in their microbiota (Bedarf et al., 2017).

### 6.6 Verrucomicrobia phylum

Bacteria from the *Verrucomicrobia* phylum have been demonstrated to possess pro-inflammatory characteristics in clinical studies (Keshavarzian et al., 2015). Moreover, the *Verrucomicrobia* phylum is present in small quantities in the typical flora of healthy individuals (Eckburg et al., 2005). However, alterations in abundance were observed in aged adults associated with low cognition scores (Manderino et al., 2017), and presented in high abundance in PD patients (Keshavarzian et al., 2015). *Akkermansia muciniphila* was found to be highly abundant in fecal samples of MS patients (Cekanaviciute et al., 2017), which could be linked with outcomes such as autoimmune encephalomyelitis and myelin damage in a mouse model of MS (Lee et al., 2011). In contrast, semi-supercentenarians with an age between 105 and 109 exhibited increased *Akkermansia* at such an advanced age, prompting further investigation into the potential role of this bacterium in healthy longevity (Biagi et al., 2016; van der Lugt et al., 2018).

## 7 Microbiota-targeted intervention for brain health

Probiotic supplementation improves the gut microbiota composition in healthy adults (Khalesi et al., 2019; Li et al., 2021). Common probiotics include *Lactobacillus*, *Bifidobacterium*, *Enterococcus*, and *Bacillus*. Probiotics can increase the levels of tryptophan-derived neurotrophic factors, and aid in either preventing or treating cognitive dysfunction (Bhattacharjee and Lukiw, 2013). Certain *Lactobacillus* and *Bifidobacterium* strains produce neurotransmitters (GABA, acetylcholine, dopamine, 5-HT) that support cognition, and neural excitatory-inhibitory balance (O’Mahony et al., 2015). Prebiotics like FOS and GOS stimulate the growth of some beneficial gut bacteria growth (Duncan and Flint, 2013).

FMT involves transferring functional bacteria from the faeces of healthy individuals into the gastrointestinal tract of patients to rebuild the gut microbiota (Li et al., 2021). Sun et al. showed that FMT from healthy mice improved motor function in PD mice by mitigating intestinal inflammation and increasing dopamine levels. The anti-inflammatory effects were achieved by reduced activity of microglia and astrocytes and increased producing SCFAs (Sun et al., 2018). Huang et al. reported that three FMT sessions improved constipation and motor symptoms in a PD patient, though tremors recurred after two months, whereas constipation was relieved even after three months (Huang et al., 2019). Additionally, FMT from healthy hamsters alleviated the ASD-like symptoms in the autism hamster model by alleviating the brain oxidative stress response. In a clinical trial with 18 children with ASD, 7–8 weeks of FMT significantly improved gastrointestinal problems and behavioural symptoms (Kang et al., 2019). Also, FMT improved the bacterial diversity by increasing the abundance of *Bifidobacterium*, *Desulfovibrio*, and *Prevotella*.

## 8 Conclusion

In contrast to the brain, the gut microbiota is accessible to direct interventions, such as prebiotics, probiotics, and antibiotics, and can be influenced by lifestyle. The concept of the MGBA emerged from extensive research that clearly demonstrated a connection between the gut and the brain. The MGBA has been associated with several neurological disorders. The advent of novel techniques for studying the microbiome has facilitated a deeper comprehension of the interrelationship between neurological disorders and the gut microbiota. The evidence is accumulating that the gut microbiome affects brain morphology, function, and behaviour, including depression, psychosis, and neurological disorders. While there is a consensus on the influence of the gut microbiota on neuroplasticity and behaviour, several questions remain enigmatic. These include the following: It would be advantageous to determine which types of neurons are influenced by signals from the gut microbiota. What is the impact of the gut microbiota on the excitation/inhibition balance in the brain? Does antibiotic-induced dysbiosis affect memory storage? A deeper comprehension of the MGBA may facilitate research into microbial-based interventions and therapeutic strategies for neurological diseases.

## Glossary

Aβ: Amyloid-beta
AChE: Acetylcholinesterase
AD: Alzheimer’s disease
ADHD: Attention-deficit/hyperactivity disorder
AEP: Asparagine endopeptidase
ALS: Amyotrophic lateral sclerosis
AMPK: Adenosine monophosphate-activated protein kinase
ASD: Autism spectrum disorder
BBB: Blood–brain barrier
BDNF: Brain-derived neurotrophic factor
CD74: Cluster of Differentiation 74
CJD: Creutzfeldt–Jakob disease
CML: N6-carboxymethyllysine
Cntnap2: Contactin-associated protein-like 2 protein
CNS: Central nervous system
COX-1: Cyclooxygenase-1
CD: Crohn’s disease
DG: Dentate gyrus
ΔFosB: Protein fosB
ENS: Enteric nervous system
FFAR: Free fatty acid receptor
FGF21: Fibroblast growth factor-21
FMT: Fecal microbiota transplantation
GF: Germ-free
GI: Gastrointestinal tract
GOS: Galacto-oligosaccharides
GYS1: Glycogen synthase 1
HDACs: Histone deacetylases
HPA: Hypothalamic–pituitary–adrenal axis
IL: Interleukins
5-LOX: 5-lipoxygenase
LDHA, LDHB: Lactate dehydrogenase-A or B
LPS: Lipopolysaccharide
LTP: Long-term potentiation
MCT1: Monocarboxylate transporter 1
MDD: Major depressive disorder
MGBA: Microbiota-gut-brain axis
MS: Multiple sclerosis
mTOR: Mammalian target of rapamycin
NMDA: N-methyl-D-aspartate
NMDAR: Anti-N-methyl-D-aspartate receptor
OLs: Oligodendrocytes
PFC: Prefrontal cortex
PFKFB3: 6-phosphofructo-2-kinase/fructose-2,6-biphosphatase 3
PrPSc: Scrapie prion protein
PUFAs: Polyunsaturated fatty acids
SCFAs: Short-chain fatty acids
SCZ: Schizophrenia
SGZ: Subgranular zone
Shank3: SH3 and multiple ankyrin repeat domains 3
SIRT-1: Sirtuin 1
TH: Tyrosine hydroxylase
TNF-α: Tumor necrosis factor-alpha
ZO-1: Zonula occludens-1
